# ﻿Examination of the generic concept and species boundaries of the genus *Erioscyphella* (Lachnaceae, Helotiales, Ascomycota) with the proposal of new species and new combinations based on the Japanese materials

**DOI:** 10.3897/mycokeys.87.73082

**Published:** 2022-02-08

**Authors:** Yukito Tochihara, Tsuyoshi Hosoya

**Affiliations:** 1 Department of Biological Sciences, Graduate School of Science, The University of Tokyo, Hongo 7-3-1, Bunkyo-ku, Tokyo 113-0033, Japan The University of Tokyo Tokyo Japan; 2 Department of Botany, National Museum of Nature and Science, 4-1-1 Amakubo, Tsukuba, Ibaraki 305-0005, Japan Department of Botany, National Museum of Nature and Science Tsukuba Japan

**Keywords:** ITS, morphology, phylogeny, species delimitation, species hypothesis, taxonomy, UNITE

## Abstract

The genus *Erioscyphella* Kirschst., which was morphologically confused with *Lachnum*, was herein examined. Based on molecular phylogenetic analyses using a combined dataset of ITS, LSU, mtSSU, and RPB2 and morphological examinations, *Erioscyphella* was distinguished from *Lachnum* and redefined by longer ascospores and the presence of apical amorphous materials and/or resinous materials equipped on hairs. Species boundaries recognized by morphology/ecology and phylogenetic analyses were cross-checked using species delimitation analyses based on DNA barcode sequences downloaded from UNITE, resulting in that species’ taxonomic problems being uncovered. Six new species (*E.boninensis*, *E.insulae*, *E.otanii*, *E.papillaris*, *E.paralushanensis*, and *E.sasibrevispora*) and two new combinations (*E.hainanensis* and *E.sinensis*) were proposed.

## ﻿Introduction

The genus *Erioscyphella* Kirschst belongs to the family Lachnaceae Raitv. (Helotiales, Ascomycota) and includes 11 species: *E.abnormis* (Mont.) Baral, Šandová & B. Perić [lectotype of *Erioscyphella* (Haines and Dumont 1984); as ‘*E.longispora* (P. Karst.) Kirschst.’ in the original description (Kirschstein 1938)], *E.alba* Ekanayaka & K.D. Hyde, *E.aseptata* Ekanayaka & K.D. Hyde, *E.bambusina* (Bres.) Kirschst., *E.brasiliensis* (Mont.) Baral, Šandová & B. Perić, *E.curvispora* B. Perić & Baral, *E.euterpes* (S.A. Cantrell & J.H. Haines) Guatim., R.W. Barreto & Crous, *E.fusiformis* (Ekanayaka & K.D. Hyde) Ekanayaka & K.D. Hyde, *E.lunata* (W.Y. Zhuang & Spooner) B. Perić & Baral, *E.lushanensis* (W.Y. Zhuang & Zheng Wang) Guatim., R.W. Barreto & Crous, and *E.sclerotii* (A.L. Sm.) Baral, Šandová & B. Perić. (Index Fungorum 2021).

*Erioscyphella* has been suggested as a monophyletic group by molecular phylogenetic analyses by Cantrell and Hanlin (1997), Hosoya et al. (2010), Perić and Baral (2014), and Guatimosim et al. (2016). However, the morphological delimitation of the genus is currently ill-defined. In the original description (Kirschstein 1938), *Erioscyphella* was misleadingly defined based on features that are not taxonomically informative, such as filiform, colored, and pigmented ascospores and lanceolate paraphyses (Korf 1978; Perić and Baral 2014). After that, in the genus *Lachnum* Retz. [type genus of Lachnaceae], species of so-called ‘long-spored *Lachnum*’, which were characterized by longer ascospores and the occurrence in tropical areas, were suggested as members of *Erioscyphella* (Haines and Dumont 1984) and have been transferred into *Erioscyphella* based on molecular phylogenetic analyses by Perić and Baral (2014) and Guatimosim et al. (2016). However, in fact, as morphology of *Erioscyphella*, including ‘long-spored *Lachnum*’, is consecutive with that of the genus *Lachnum* especially regarding the ascospore length and shape of ectal excipular cells (Haines and Dumont 1984), the morphological delimitation of *Erioscyphella* has not been sufficiently discussed. Since much more potential species are thought to be included in *Erioscyphella*, its morphological concept must be discussed and updated based on a wider size of taxon sampling.

In the present study, the authors aimed to: a) clarify the generic boundaries of *Erioscyphella* using molecular and morphological/ecological data, and b) propose new species or new combinations based on more objectively defined species boundaries. To reach our first goal, we used specimens from the herbarium of the National Museum of Nature and Science (TNS) (Tsukuba, Japan) as most of them were accompanied by culture and/or DNA extracts. In TNS, only three identified species of *Erioscyphella* were recognized (*E.abnormis*, *E.brasiliensis*, and *E.sclerotii*); however, we presumed that many unidentified species of *Erioscyphella* were housed therein. To reach our second goal, for species recognition, we tested DNA barcoding using the internal transcribed spacer region of nuclear ribosomal DNA (ITS), widely accepted as fungal DNA barcode (Begerow et al. 2010; Schoch et al. 2012; Hosoya 2021). ITS-based species boundaries were explored based on multiple methods, and the results were compared to species boundaries based on morphology, ecology, and phylogenetic relationships.

## ﻿Materials and methods

### ﻿Taxon sampling

In TNS, specimens labeled as *Erioscyphella* were initially searched, and closely related specimens to *Erioscyphella* were searched based on the sequence similarities of ITS. Selected specimens were tentatively identified based on morphology following Dennis (1954), Haines (1980), Haines and Dumont (1984), Spooner (1987), and Perić and Baral (2014).

### ﻿Morphological observation, DNA extraction, and sequencing

Micromorphology was examined using cotton blue (CB) dissolved in lactic acid (LA) (CB/LA; 0.5 g CB and 99.5 mL LA) as a mounting fluid. To check the ascal apex iodine reaction, Melzer’s reagent (MLZ; 0.5 g I_2_, 1.5 g KI, 20 g chloral hydrate, and 20 g water) was initially used without KOH pretreatment, and Lugol’s iodine (IKI; 1 g I_2_ and 1 g KI, and 100 mL H_2_O) and MLZ with 3% KOH pretreatment were used when necessary. World Geodetic System 84 was used for the geographic coordinates. URLs herein shown were accessed on April 15, 2021, except for GBIF website accessed on Feb 10, 2020.

DNA was extracted from cultivated isolates in 2% malt extract broth (MEB) using the modified cetyltrimethylammonium bromide (CTAB) method (Hosaka and Castellano 2008; Tochihara and Hosoya 2019). When isolates are not available, DNA was extracted directly from a crushed apothecium using DNA extraction buffer following Tochihara and Hosoya (2019). The isolates from which DNA extracted were deposited in the NITE National Biological Resource Center (NBRC) (Kisarazu, Japan), except for isolates with restriction on transition by Japanese laws and those unavailable because of contracts with private companies.

Polymerase chain reaction (PCR) was used to amplify the following regions: ITS (= ITS1-5.8S-ITS2), the partial large subunit nuclear ribosomal RNA gene (LSU), the partial mitochondrial small subunit (mtSSU), and section ‘6–7’ of the second largest subunit of the nuclear RNA polymerase II gene (RPB2). Primer pairs for PCR reactions of ITS, LSU and mtSSU were ITS1F (5’–CTTGGTCATTTAGAGGAAGTAA–3’) (Gardes and Bruns 1993) or ITS1 (5’–TCCGTAGGTGAACCTCGGG–3’) (White et al. 1990) and ITS4 (5’–TCCTCCGCTTATTGATATGC–3’) (White et al. 1990), LR0R (5’–ACCCGCTGAACTTAAGC–3’) and LR5 (5’–TCCTGAGGGAAACTTCG–3’) (Vilgalys and Hester 1990), and mrSSU1 (5’–AGCAGTGAGGAATATTGGTC–3’) and mrSSU3R (5’–ATGTGGCACGTCTATAGCCC–3’) (Zoller et al. 1999) respectively. The PCR program consisted of an initial denaturation at 95 °C for 3 min, followed by 30 cycles of 94 °C for 35 s, 51 °C for 30 s, and 72 °C for 1 min, and a final extension at 72 °C for 10 min. When appropriate PCR products were not obtained, a modified PCR program was applied first, and then alternative primer pairs were tested. For RPB2, an alternative forward primer fRPB2-5F (5’–GAYGAYMGWGATCAYTTYGG–3’) (Liu et al. 1999) or RPB2-P6Fa (5’–TGGGGRYTK GTBTGYCCKGCHGA–3’) (Hansen et al. 2005) and a reverse primer bRPB2-7.1R2 (5’–CCCATNGCYTGYTTVCCCATDGC–3’) (modified from bRPB2-7.1R) (Matheny 2005; Matheny et al. 2007; Gelardi et al. 2015) were used.

Sequencing was conducted on an ABI PRISM 3500xL Genetic Analyzer (Applied Biosystems; Thermo Fisher Scientific, Waltham, MA, USA) with a BigDye Terminator 3.1 Cycle Sequencing Kit (Applied Biosystems). The obtained sequences were assembled using ATGC 7 (Genetyx, Tokyo, Japan). Assembled sequences were deposited in the International Nucleotide Sequence Database Collaboration (INSDC) via the DNA Data Bank of Japan (DDBJ), and acquired INSDC accession numbers. Assembled ITS sequences were also deposited in the UNITE database (https://unite.ut.ee/) via the PlutoF workbench (https://plutof.ut.ee/) (Abarenkov et al. 2010) and acquired UNITE accession numbers.

### ﻿Phylogenetic analyses

The specimens obtained from TNS were included in the phylogenetic analyses as candidate members of *Erioscyphella* (‘‡’ in Table 1). From other genera of the family Lachnaceae, four species of *Lachnum*, two species of *Albotricha*, *Brunnipila*, *Capitotricha*, *Dasyscyphella*, *Incrucipulum*, and *Lachnellula*, and one species of *Neodasyscypha* and *Proliferodiscus* were used (‘†’ in Table 1). Among the eight genera, seven of them (except *Proliferodiscus*) included type species. Three species of Helotiales were selected as outgroups following Tochihara and Hosoya (2019) (Table 1).

**Table 1.** Taxa analyzed in the phylogenetic analyses.

Specimen no. (TNS-F-)	Taxon^|^	Collection site	Collected Date	Host plants and parts	Strain no. (NBRC^¶^)	UNITE/GenBank accession no.^#^			
						ITS	LSU	mtSSU	RPB2
†16740	*Albotrichaacutipila* (P. Karst.) Raitv.	Japan, Nagano, Ueda, Sugadaira Montane Research Center	2006-06-17	culm of unidentified bamboo	104380	AB481234	LC438571	LC431751	AB481354
†16497	*Albotrichaalbotestacea* (Desm.) Raitv.	Japan, Nagano, Ueda, Sugadaira Montane Research Center	2005-05-18	culm of *Miscanthussinensis*	101346	AB481235	LC424943	LC431747	AB481340
†16635	*Brunnipilafuscescens* (Pers.) Baral	Japan, Gunma, Higashi-Agatsuma	2006-04-27	leaf of unidentified tree	104365	AB481255	LC424945	LC431750	AB481348
†16690	*Brunnipilapseudocannabina* (Raitv.) Tochihara, Sasagawa & Hosoya	JAPAN, Akita, Kosaka	2006-05-26	stem of unidentified herb	104374	AB481272	LC533520	LC533522	LC533521
†65670	*Capitotrichabicolor* (Bull.) Baral	SWITZERLAND, Filisur	2016-06-06	twig of *Prunusspinosa*	(FC-6101)	LC424834	LC424942	LC533244	LC425011
†65752	*Capitotricharubi* (Bres.) Baral	SWITZERLAND, Saicourt	2016-06-04	twig of *Rubusidaeus*	(FC-6075)	LC438560	LC438573	LC533243	LC440395
†16439	*Dasyscyphellalongistipitata* Hosoya	JAPAN, Kanagawa, Yamakita	2005-04-17	cupule of *Faguscrenata*	101335	AB481239	LC424947	LC533228	AB481331
†16527	*Dasyscyphellamontana* Raitv.	Japan, Nagano, Ueda, Sugadaira Montane Research Center	2005-05-21	wood of unidentified tree	102336	AB481242	LC438577	LC533241	AB481336
‡16556	*Erioscyphellaabnormis* (Mont.) Baral, Šandová & B. Perić	Japan, Oita, Kokonoe	2005-05	wood of unidentified tree	114449	UDB0779051	LC533153	LC533257	LC533198
‡16582	*Erioscyphellaabnormis* (Mont.) Baral, Šandová & B. Perić	Japan, Kanagawa, Yamakita	2005-07-02	wood of unidentified tree	104360	AB481249	LC533176	LC533233	LC533199
‡16606	*Erioscyphellaabnormis* (Mont.) Baral, Šandová & B. Perić	Japan, Kanagawa, Yamakita	2005-07-03	wood of unidentified tree	114450	UDB0779053	LC533154	LC533258	LC533200
‡16609	*Erioscyphellaabnormis* (Mont.) Baral, Šandová & B. Perić	Japan, Kanagawa, Yamakita	2005-07-03	wood of *Cephalotaxusharringtonia*	101350	††AB705234	LC533175	LC533256	LC533184
‡16639	*Erioscyphellaabnormis* (Mont.) Baral, Šandová & B. Perić	Japan, Ibaraki, Tsukuba Botanical Garden	2006-05-01	twig of unidentified tree	114451	UDB0779054	LC533155	LC533259	LC533201
‡25579	*Erioscyphellaabnormis* (Mont.) Baral, Šandová & B. Perić	Japan, Tokyo, Hongo	2009-05-25	twig of unidentified tree	(FC-1887)	UDB0779057	LC533146	LC533250	LC533191
‡32163	*Erioscyphellaabnormis* (Mont.) Baral, Šandová & B. Perić	Japan, Kanagawa, Odawara	2010-05-14	twig of unidentified tree	114456	UDB0779062	LC533158	LC533260	LC533203
‡38452	*Erioscyphellaabnormis* (Mont.) Baral, Šandová & B. Perić	Japan, Gunma, Naganohara	2013-06-27	wood of unidentified tree	114463	††UDB0779069	LC533171	LC533262	LC533210
‡46416	*Erioscyphellaabnormis* (Mont.) Baral, Šandová & B. Perić	Taiwan, Taipei	2012-04-15	wood of unidentified tree	(FC-2906)	UDB0779067	LC533132	LC533277	LC549671
‡46841	*Erioscyphellaabnormis* (Mont.) Baral, Šandová & B. Perić	Japan, Gifu, Gujo	2012-05-28	wood of unidentified tree	114462	UDB0779086	LC533170	LC533279	LC533209
‡61773	*Erioscyphellaabnormis* (Mont.) Baral, Šandová & B. Perić	Japan, Kanagawa, Yokohama	2015-04-01	twig of unidentified tree	114464	††UDB0779074	LC533137	LC533264	LC533211
‡61931	*Erioscyphellaabnormis* (Mont.) Baral, Šandová & B. Perić	Japan, Kanagawa, Zushi	2015-04-16	wood of unidentified tree	114466	UDB0779072	LC533139	LC533266	LC533213
‡80478	*Erioscyphellaabnormis* (Mont.) Baral, Šandová & B. Perić	Japan, Shizuoka, Oyama	2017-06-26	twig of unidentified tree	113934	LC424837	LC424949	LC533283	LC425009
†26520	*Erioscyphellaboninensis* Tochihara & Hosoya	Japan, Tokyo, Chichijima Island	2009-06-28	trunk of unidentified tree	114447	UDB0779049	LC533151	LC533254	LC533196
‡46419	*Erioscyphellabrasiliensis* (Mont.) Baral, Šandová & B. Perić	Taiwan, Taipei	2012-04-20	wood of unidentified tree	(FC-2910)	UDB0779068	LC533133	LC533278	LC549672
‡35049	*Erioscyphellahainanensis* (W.Y. Zhuang and Zheng Wang) Hosoya and Tochihara (←*Lachnumhainanense* W.Y. Zhuang and Zheng Wang)	Japan, Niigata, Minamiuonuma	2010-05-14	leaf of *Quercusglauca*	114457	UDB0779064	LC533168	LC533274	LC533205
‡35056	*Erioscyphellahainanensis* (W.Y. Zhuang and Zheng Wang) Hosoya and Tochihara (←*Lachnumhainanense* W.Y. Zhuang and Zheng Wang)	Japan, Niigata, Minamiuonuma	2010-05-14	leaf of *Quercusserrata*	114458	UDB0779065	LC533169	LC533275	LC533206
‡61775	*Erioscyphellahainanensis* (W.Y. Zhuang and Zheng Wang) Hosoya and Tochihara (←*Lachnumhainanense* W.Y. Zhuang and Zheng Wang)	Japan, Kanagawa, Hiratsuka	2015-04-12	leaf of *Quercusmyrsinifolia*	114465	UDB0779071	LC533138	LC533265	LC533212
‡61941	*Erioscyphellahainanensis* (W.Y. Zhuang and Zheng Wang) Hosoya and Tochihara (←*Lachnumhainanense* W.Y. Zhuang and Zheng Wang)	Japan, Kanagawa, Kamakura	2015-04-24	leaf of *Quercusglauca*	112569	UDB0779073	LC533140	LC533280	LC533214
‡65722	*Erioscyphellahainanensis* (W.Y. Zhuang and Zheng Wang) Hosoya and Tochihara (←*Lachnumhainanense* W.Y. Zhuang and Zheng Wang)	Japan, Gunma, Midori	2016-04-24	leaf of *Quercusserrata* subsp. *Mongolicoides*	114469	UDB0779076	LC533142	LC533281	LC533215
‡80356	*Erioscyphellahainanensis* (W.Y. Zhuang and Zheng Wang) Hosoya and Tochihara (←*Lachnumhainanense* W.Y. Zhuang and Zheng Wang)	Japan, Kanagawa, Hiratsuka	2017-05-18	leaf of *Quercusglauca*	114470	UDB0779077	LC533172	LC533282	LC533186
‡80371	*Erioscyphellahainanensis* (W.Y. Zhuang and Zheng Wang) Hosoya and Tochihara (←*Lachnumhainanense* W.Y. Zhuang and Zheng Wang)	Japan, Kanagawa, Hiratsuka	2017-05-18	leaf of *Castanopsissieboldii*	114472	UDB0779078	LC533135	LC533246	LC533188
‡26500	*Erioscyphellainsulae* Tochihara & Hosoya	Japan, Tokyo, Hahajima Island	2009-06-24	wood of unidentified tree	114445	UDB0779060	LC533149	LC533252	LC533194
‡39720	*Erioscyphellainsulae* Tochihara & Hosoya	Japan, Okinawa, Iriomote Island	2011-06-12	bark of unidentified tree	114459	UDB0779063	LC533177	LC533261	LC533207
‡61920	*Erioscyphellaparalushanensis* Tochihara & Hosoya	Japan, Shizuoka, Atami	2015-06-08	culm of *Pleioblastusargenteostriatus*	114468	††UDB0779075	LC533141	LC533267	LC533220
†81472	*Erioscyphellaotanii* Tochihara	Japan, Hokkaido, Horonobe, Teshio Experimental Forest, Hokkaido University	2018-07-11	leaf of *Sasasenanensis*	114476	UDB0779085	LC533179	LC533286	||LC533226
‡81272	*Erioscyphellapapillaris* Tochihara	Japan, Gunma, Minakami	2017-07-16	leaf of unidentified bamboo	113937	UDB0779081	LC533161	LC533285	LC533204
‡80399	*Erioscyphellasasibrevispora* Tochihara & Hosoya	Japan, Gunma, Higashi-Agatsuma	2017-06-06	sheath of *Sasaveitchii*	―	UDB0779082/LC669470	LC533173	LC533268	LC533216
‡81401	*Erioscyphellasasibrevispora* Tochihara & Hosoya	Japan, Hokkaido, Tomakomai	2018-06-16	culm of *Sasanipponica*	114475	UDB0779084/LC669472	LC533174	LC533269	LC533217
‡26492	*Erioscyphellasclerotii* (A.L. Sm.) Baral, Šandová & B. Perić	Japan, Tokyo, Hahajima Island	2009-06-24	wood of unidentified tree	114448	UDB0779050/LC669438	LC533152	LC533255	LC533197
‡38480	*Erioscyphellasclerotii* (A.L. Sm.) Baral, Šandová & B. Perić	Taiwan, Wulai	2013-07-12	twig of unidentified tree	(FC-5208)	††UDB0779070	LC533134	LC533263	LC549673
‡16838	*Erioscyphellasinensis* (Z.H. Yu and W.Y. Zhuang) Sasagawa, Tochihara & Hosoya (←Lachnummapirianumvar.sinense Z.H. Yu and W.Y. Zhuang)	Japan, Ibaraki, Tsukuba Botanical Garden	2007-06-15	leaf of unidentified broad-leaved tree	104389	AB481280	LC533164	LC533235	AB481364
‡80354	*Erioscyphellasinensis* (Z.H. Yu and W.Y. Zhuang) Sasagawa, Tochihara & Hosoya (←Lachnummapirianumvar.sinense Z.H. Yu and W.Y. Zhuang)	Japan, Kanagawa, Manazuru	2017-05	leaf of *Castanopsissieboldi*	114471	UDB0779083/LC669471	LC533143	LC533245	LC533187
‡16841	*Erioscyphellasinensis* (Z.H. Yu and W.Y. Zhuang) Sasagawa, Tochihara & Hosoya (←Lachnummapirianumvar.sinense Z.H. Yu and W.Y. Zhuang)	Japan, Ibaraki, Mt. Tsukuba	2007-06-23	leaf of unidentified broad-leaved tree	104390	AB481281	LC533157	LC533236	LC533218
‡32161	*Erioscyphellasinensis* (Z.H. Yu and W.Y. Zhuang) Sasagawa, Tochihara & Hosoya (←Lachnummapirianumvar.sinense Z.H. Yu and W.Y. Zhuang)	Japan, Kanagawa, Odawara	2010-05-14	leaf of *Quercusmyrsinifolia*	113715	UDB0779061/LC669449	LC533167	LC533273	LC533219
‡16837	*Erioscyphellasinensis* (Z.H. Yu and W.Y. Zhuang) Sasagawa, Tochihara & Hosoya (←Lachnummapirianumvar.sinense Z.H. Yu and W.Y. Zhuang)	Japan, Ibaraki, Tsukuba Botanical Garden	2007-06-15	leaf of unidentified broad-leaved tree	114452	UDB0779055/LC669443	LC533156	LC533272	LC533202
†81520	*Incrucipulumciliare* (Schrad.) Baral	Japan, Shizuoka, Shizuoka	2018-08-18	leaf of Quercusmongolicasubsp.crispula	113941	LC438566	LC438583	LC533284	LC438596
†17632	*Incrucipulumlongispineum* Sasagawa & Hosoya	Japan, Miyagi, Sendai	2006-07-29	leaf of *Lyoniaovalifolia*	102347	AB481256	LC438579	LC533234	AB481362
†81248	*Lachnellulacalyciformis* (Batsch) Dharne	Japan, Hokkaido, Engaru	2017-07-12	twig of *Abiessachalinensis*	113935	LC438561	LC438574	LC533247	LC438590
†16529	*Lachnellulasuecica* (de Bary ex Fuckel) Nannf.	Japan, Nagano, Ueda, Sugadaira Montane Research Center	2005-05-21	twig of *Larixkaempferi*	101348	AB481248	LC424944	LC533231	AB481341
†16494	*Lachnumasiaticum* (Y. Otani) Raitv.	Japan, Nagano, Ueda, Sugadaira Montane Research Center	2005-05-18	culm of unidentified bamboo	101341	AB481251	LC533162	LC533229	AB481334
‡17249	*Lachnummapirianum* (Pat. & Gaillard) M.P. Sharma	Malaysia, Gerik	2004-09-07	leaf of unidentified tree	―	UDB0779088/LC669476	LC533182	―	LC533223
‡17245	*Lachnummapirianum* (Pat. & Gaillard) M.P. Sharma	Malaysia, Gerik	2004-09-07	leaf of unidentified tree	―	UDB0779087/LC669475	LC533181	―	LC533222
‡16442	Lachnumnovoguineensevar.yunnanicum W.Y. Zhuang	Japan, Nagano, Ueda, Sugadaira Montane Research Center	2005-05-18	culm of unidentified bamboo	102339	AB481270	LC533163	LC533232	AB481342
‡16642	Lachnumnovoguineensevar.yunnanicum W.Y. Zhuang	Japan, Ibaraki, Mt. Tsukuba	2006-05-02	culm of unidentified bamboo	104368	AB481271	LC533165	LC533227	§§LC533225
‡11197	*Lachnumpalmae* sensu lato (←*Lachnumpalmae* (Kanouse) Spooner)	Japan, Shizuoka, Shimoda	2004-07-26	leaf of Livistonachinensisvar.subglobosa	106495	UDB0779047/LC669435	LC533166	LC533248	LC533185
‡13500	*Lachnumpalmae* sensu lato (←*Lachnumpalmae* (Kanouse) Spooner)	Japan, Kagoshima, Yakushima Island	2005-10-19	leaf of Livistonachinensisvar.subglobosa	114441	††LC425039/UDB779046	LC429382	LC533240	‡‡LC431718
‡17567	*Lachnumpalmae* sensu lato (←*Lachnumpalmae* (Kanouse) Spooner)	New Zealand	2005-05-28	leaf of unidentified palm	―	UDB0779089/LC669477	LC533183	LC533288	―
‡24588	*Lachnumpalmae* sensu lato (←*Lachnumpalmae* (Kanouse) Spooner)	Japan, Kagoshima, Amami-Oshima	2009-02-24	leaf of Livistonachinensisvar.subglobosa	114442	UDB0779052/LC669440	LC533144	LC533270	LC533190
‡24600	*Lachnumpalmae* sensu lato (←*Lachnumpalmae* (Kanouse) Spooner)	Japan, Kagoshima, Amami-Oshima	2009-02-25	leaf of Livistonachinensisvar.subglobosa	114443	UDB0779056/LC669444	LC533145	LC533249	||LC533224
‡26161	*Lachnumpalmae* sensu lato (←*Lachnumpalmae* (Kanouse) Spooner)	Japan, Tokyo, Chichijima Island	2009-06-27	leaf of *Livistonaboninensis*	114446	UDB0779048/LC669436	LC533150	LC533253	LC533195
‡26172	*Lachnumpalmae* sensu lato (←*Lachnumpalmae* (Kanouse) Spooner)	Japan, Tokyo, Kita-Iwojima Island	2009-06-17	leaf of Livistonachinensisvar.subglobosa	(FC-1935)	UDB0779058/LC669446	LC533147	LC533251	LC533192
‡26185	*Lachnumpalmae* sensu lato (←*Lachnumpalmae* (Kanouse) Spooner)	Japan, Tokyo, Kita-Iwojima Island	2009-06-18	leaf of Livistonachinensisvar.subglobosa	114444	UDB0779059/LC669447	LC533148	LC533271	LC533193
‡39729	*Lachnumpalmae* sensu lato (←*Lachnumpalmae* (Kanouse) Spooner)	Japan, Okinawa, Iriomote Island	2011-06-13	leaf of Livistonachinensisvar.subglobosa	114460	UDB0779066/LC669454	LC533178	LC533276	LC533208
†16501	*Lachnumpudibundum* (Quél.) J. Schröt.	Japan, Nagano, Ueda, Sugadaira Montane Research Center	2005-05-18	wood of unidentified tree	102335	AB481259	LC533160	LC533230	AB481335
†81229	*Lachnumrachidicola* J.G. Han, Raitv. & H.D. Shin	Japan, Hokkaido, Tomakomai, Tomakomai Experimental Forest	2017-08-09	petiole of *Juglans* sp.	114473	UDB0779079/LC669467	LC533136	―	LC533189
†16583	*Lachnumvirgineum* (Batsch) P. Karst.	Japan, Kanagawa, Yamakita	2005-07-02	wood of unidentified tree	104358	AB481268	AB926119	LC431748	AB481343
†65625	*Neodasyscyphacerina* (Pers.) Spooner	Switzerland, Saicourt	2016-06-08	twig of *Crataegus* sp.	(FC-6068)	LC424836	LC424948	LC533242	LC425013
†17436	*Proliferodiscusalboviridis* (Sacc.) Spooner	Japan, Ibaraki, Tsukuba Botanical Garden	2006-07-08	wood of unidentified tree	108594	LC438558	LC533159	LC533239	LC425014
§17909	*Hyaloscyphaspiralis* (Velen.) J.G. Han, Hosoya & H.D. Shin	Japan, Kumamoto, Kikuchi	2005-10-10	wood of unidentified tree	108585	††LC438602	LC438604	LC533237	LC438606
§16472	*Hymenoscyphusvaricosporoides* Tubaki	Japan, Ibaraki, Kasumigaura	2005-05-05	wood of unidentified tree	104355	AB926052	LC424952	LC431746	AB481329
§18014	*Urceolellacarestiana* (Rabenh.) Dennis	Japan, Iwate, Hanamaki	2006-05-23	stem of *Parathelypterisnipponica*	108588	††LC438603	LC438605	LC533238	LC438607

† Lachnaceae except for Erioscyphella and its potential species tentatively identified based on morphology ‡ Erioscyphella or its potential species tentatively identified based on morphology § Outgroup | Original taxon name labeled on the specimen is shown enclosed by “(←)” and is only shown when it is different from a name determined in this study. ¶ Cultures not donated in NBRC is beginning with “FC-”, local suffix in TNS. ‘―’ represents no culture exist and DNA was extracted from apothecia. # UNITE accession no. is beginning with ‘UDB’. GenBank accession no. is beginning with ‘AB’ or ‘LC’. †† Primer pair ITS1 and ITS4 was used. In ITS sequences without notes (††), primer pair ITS1F and ITS4 was used. ‡‡ Primer pair fRPB2-5F and RPB2-P7R was used. §§ Primer pair RPB2-P6Fa and bRPB2-7.1R2 was used. || Primer pair RPB2-P6Fa and RPB2-P7R was used. In RPB2 sequences without any notes (‡‡, §§, ||), primer pair RPB2-P6F and RPB2-P7R was used.

A concatenated dataset of ITS, LSU, mtSSU, and RPB2 was used in the phylogenetic analyses. Each region was aligned separately using MAFFT 7 (Katoh and Standley 2013). The Q-INS-i option was used for ITS, LSU, and mtSSU to accommodate the secondary structures of RNA, and the G-INS-1 option was used for RPB2 to assume global alignment using the entire region. The aligned sequences were edited manually using BioEdit 7.0.5.2 (Hall 1999).

Phylogenetic conflicts among gene partitions were checked before the phylogenetic analyses using the concatenated matrix. Maximum likelihood (ML) trees with 1,000 bootstrap replications (Felsenstein 1985) using the ITS, LSU, mtSSU, and RPB2 datasets separately were constructed using MEGA X (Kumar et al. 2018) with the GTR+G model; branches with bootstrap values > 70% were compared among trees. For mtSSU and RPB2, specimens containing missing data were excluded from the analyses.

The concatenated dataset was analyzed using ML, maximum parsimony (MP), and Bayesian inference (BI). For the ML and BI analyses, substitution models were estimated for each partition (ITS, LSU, mtSSU, and each codon position of RPB2) based on Akaike’s information criterion (AIC) (Akaike 1974) using Modeltest-NG 0.1.6 (Darriba et al. 2019).

ML tree search (Felsenstein 1984) and bootstrapping (Felsenstein 1985; Lemoine et al. 2018) was performed using RAxML-NG 0.9.0 (Kozlov et al. 2019) with 1,000 bootstrap replications under the substitution model SYM+I+G4 for ITS, TIM1+I+G4 for LSU, TPM1uf+I+G4 for mtSSU and RPB2 third codon position, GTR+I+G4 for RPB2 first codon position, and TPM3uf+I+G4 for RPB2 second codon position. Sequence matrix containing missing data typically yield multiple trees residing on a phylogenetic terrace (Sanderson et al. 2011; Biczok et al. 2018). Therefore, we checked if the best-scored-tree did not lie on a terrace using the Python tool called ‘terraphy’ implemented in RAxML-NG 0.9.0.

MP analysis was conducted using PAUP* 4.0a 167 (Swofford 2002). All substitutions were treated as unordered and of equal weights. All gaps were treated as missing data. A heuristic parsimony search was carried out with 1,000 replicates of random step addition, with a tree bisection reconnection (TBR) branch swapping algorithm, Multrees option on, Steepest descent modification option on, and branch collapse option set to MinBrlen. Bootstrap values (MPBP; Felsenstein 1985) were estimated from 1,000 replicates of heuristic searches, with random taxon addition, TBR branch swapping, and Multrees options off.

**Figure 1.**
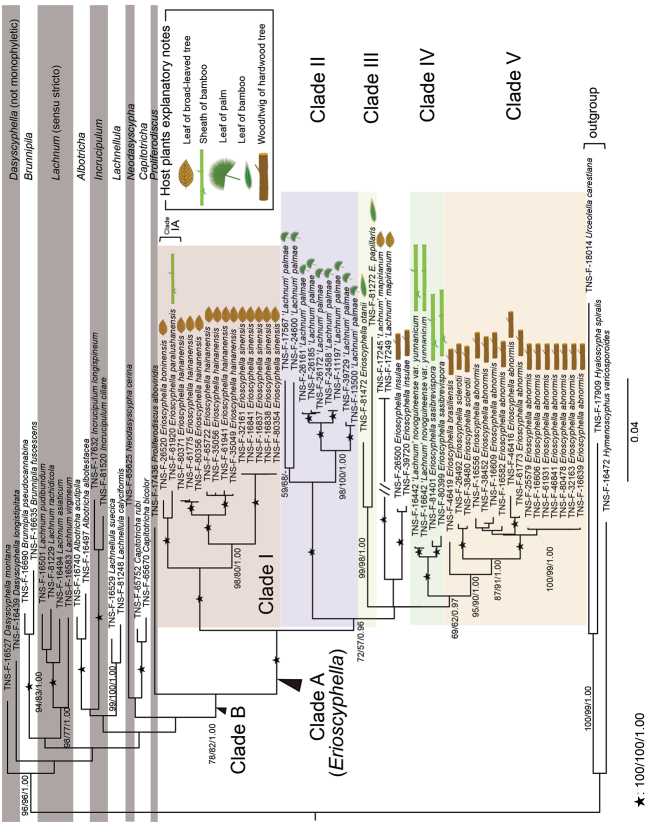
ML best-scored phylogenetic tree based on the concatenated dataset of ITS, LSU, mtSSU, and RPB2 constructed using RAxML-NG. MLBP/MPBP/BPP are represented on branches in this order. In MLBP/MPBP < 50% or BPP < 0.95, a hyphen appears. No evaluation values are shown on branches when MLBP and MPBP < 50% and BPP < 0.95. The branch of a clade TNS-F-17245 + 17249 to its most recent common ancestor is only one-third of the intended length due to space limitation.

BI analysis was based on MrBayes 3.2.7a (Ronquist et al. 2012) under the substitution model SYM+I+G4 for ITS, GTR+I+G4 for LSU and RPB2 first codon positions, HKY+I+G4 for mtSSU and RPB2 third codon positions, and F81+I for RPB2 second codon position. Two separate Metropolis-Coupled Markov Chains of Monte Carlo (MCMCMC) ran simultaneously starting from random trees for 20 million generations, and trees were sampled every 500 generations. The average standard deviation of split frequencies (ASDSF) and effective sample size (ESS) were checked using Tracer 1.7.1 (Rambaut 2018a) as an indication of convergence. Using post-burn-in trees, a 50% majority rule consensus tree was generated, and Bayesian posterior probabilities (BPP) were calculated to evaluate node supports. Trees were visualized using FigTree 1.4.4 (Rambaut 2018b) based on the ML, MP, and BI analyses respectively. Branches with MLBP and MPBP > 90% and BPP > 0.95 were regarded as strongly supported.

**Figure 2.**
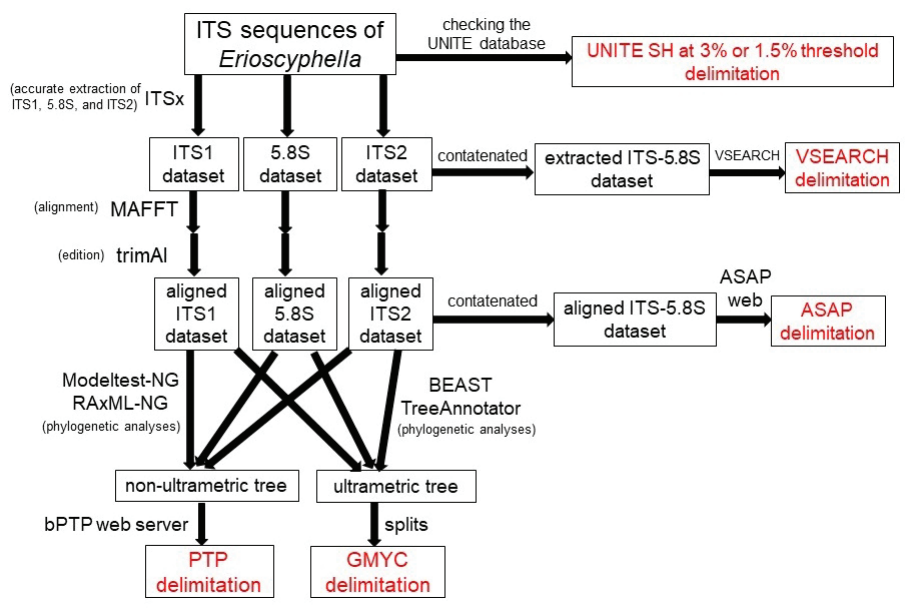
Diagrammatic representation showing the species delimitation analyses using ITS sequences.

ITS-based species delimitation analyses (Fig. 2)

To maximize the number of ITS sequences, we used the UNITE Species Hypotheses (SH) system provided by the UNITE database (Kõljalg et al. 2013; Nilsson et al. 2015; GBIF 2018; Kõljalg et al. 2020). In the UNITE SH system, all fungal ITS sequences are periodically divided into species-level clusters (species hypothesis; SH) at optional sequence-distance thresholds (0%–3% in 0.5% steps), each of which is assigned to a unique UNITE SH code represented by a digital objective identifier (DOI) accessible from internet (Kõljalg et al. 2016, 2020; Nilsson et al. 2015).

Based on the UNITE SH system, we collected ITS sequences of *Erioscyphella* in the following process: a) selectivity of closely related sequences: for every ITS sequence newly obtained from TNS specimens (= query sequences, 49 sequences), UNITE SH code at the 3% threshold value were searched in the UNITE database to gather sequences in wider scope, and all sequences within the UNITE SH code were downloaded. b) selectivity based on taxon names: using the UNITE search page, ITS sequences named *Erioscyphella* were searched, because only closely related sequences to query sequences are filtered under the a) criterion. Sequences with synonyms of *Eriosyphella* species were also searched, because the UNITE lookup function is not supported by any backbone taxonomies to integrate synonyms. Sequences satisfying criterion a) or b) were downloaded for ITS-based species recognition. The obtained ITS sequences were clustered into SHs based on an OTU clustering method, hierarchical clustering method, and two coalescent-based methods. For all ITS sequences, ITS1, 5.8S, and ITS2 regions were extracted using ITSx (Nilsson et al. 2010) to construct an accurate ITS dataset, because the inclusion of segments of adjacent regions (such as a small subunit of 18S rRNA or LSU) may decrease the accuracy of the calculation of ITS distances (Nilsson et al. 2010). OTU clustering was executed using VSEARCH v2.17.2 (Rognes et al. 2016) implemented in the Qiime 2 microbiome analysis platform (Bolyen et al. 2019).

The concatenated dataset of extracted ITS1, 5.8S, and ITS2 was incorporated into VSEARCH, and OTU clustering at 97% and 98.5% similarity thresholds were performed using the ‘-cluster_fast’ option. Hierarchical clustering based on pairwise sequence distances was executed using the Assemble Species by Automatic Partitioning (ASAP) method (Puillandre et al. 2021). The datasets of extracted ITS1, 5.8S, and ITS2 were separately aligned using MAFFT 7 under the Q-INS-i option and edited using trimAl v1.2 (Capella-Gutiérrez et al. 2009) under the ‘-gappyout’ option. The concatenated dataset of the three aligned partitions was analyzed using ASAP web (https://bioinfo.mnhn.fr/abi/public/asap/asapweb.html). Jukes-Cantor (JC69) was selected as a substitution model for computing pairwise distances of sequences. As phylogeny-based species delimitation methods, the generalized mixed Yule-coalescent (GMYC) model (Pons et al. 2006; Fujisawa and Barraclough 2013) and the Poisson Tree Processes (PTP) model (Zhang et al. 2013) were used. In both models, speciation (species-level differentiation) and coalescence (population-level differentiation) are identified based on the length of phylogenetic trees. GMYC requires the use of phylogenetic trees following the molecular clock model (= ultrametric tree) because it detects transition points from speciation to coalescence focusing on the time axis, while PTP does not require ultrametric tree as it focuses on the number of nucleotide substitutions. Ultrametric trees were estimated using BEAST v2.6.3. (Bouckaert et al. 2019). The ITS dataset was divided into ITS1, 5.8S, and ITS2, and suitable substitution models GTR+G for ITS1 and JC+G for 5.8S and ITS2 estimated using Modeltest-NG 0.1.6. were applied. To estimate branch length, a Yule model and a relaxed clock with a log-normal distribution were selected. MCMC chains were run for 1.5×10^8^ generations and sampled every 1,000 generations. After each run, convergence was checked using Tracer 1.7.1, and the first 10% were discarded as burn-in. A consensus tree was generated using TreeAnnotator v1.10.4 in BEAST package, from 150,000 generated trees except for the first 10% regarded as burn-in. A single-threshold species delimitation analysis based on GMYC was conducted using the R package ‘splits’ (Fujisawa and Barraclough 2013).

For the species delimitation analyses using PTP, an unrooted ML phylogenetic tree was constructed using RAxML-NG 0.9.0. The analysis used ITS1, 5.8S, and ITS2 partitions, aligned as previously described, under the substitution models TIM2+G4 for ITS1, TPM2+I+G4 for 5.8S, and GTR+I+G4 for ITS2, estimated using Modeltest-NG 0.1.6. based on the AIC. The species delimitation analysis was executed using the generated ML best-scored tree with the bPTP web server (https://species.h-its.org/). The MCMC run was set to 500,000 generations and burn-in rate was set to 0.1. The convergence of MCMC runs was visually checked. In ML and Bayesian results, a result generating fewer SHs was adopted to avoid excessive species division.

SHs generated in the species delimitation analyses and the UNITE SHs at 3% and 1.5% threshold values were compared with one another.

### ﻿Species recognition

In the present study, we initially recognized species boundaries based on the two criteria:

1. Forming a monophyletic group in the phylogenetic analyses based on multigene data (Fig. 1).
2. Members can be distinguished based on morphological and/or common ecological features (such as host plants).

**Figure 3.**
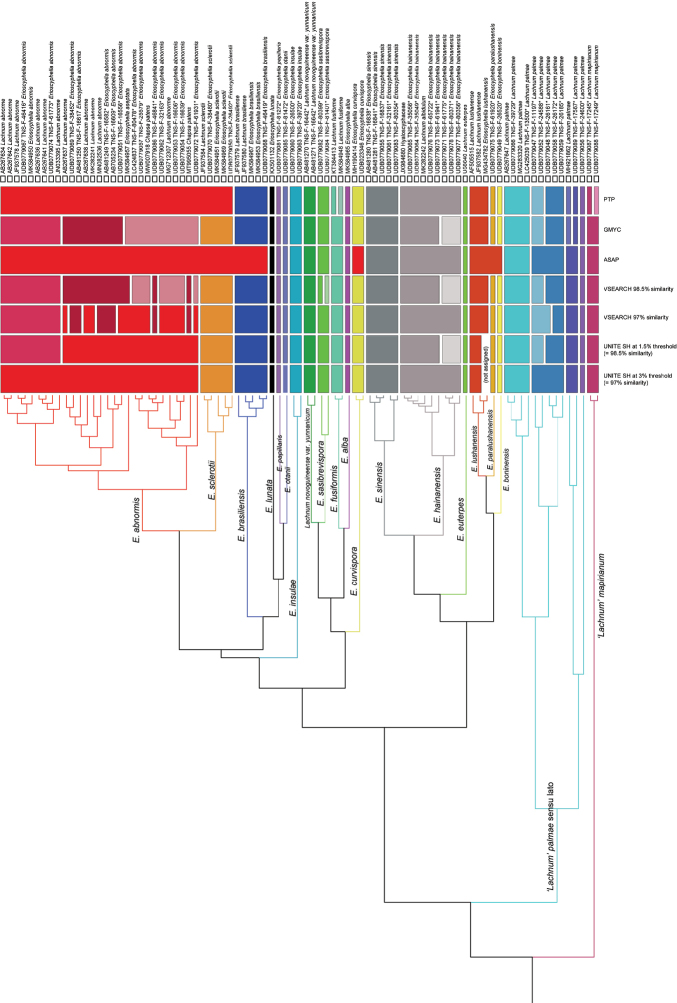
Species delimitation analyses using ITS sequences of *Erioscyphella* and its potential members. Clusters based on UNITE SH at 3% and 1.5% threshold values at UNITE v8.2, VSEARCH at 97% and 98.5% threshold values, ASAP, GMYC, and PTP are displayed. Schematic phylogenetic relationships are shown using the ultrametric tree constructed for the GMYC analysis. The taxon names shown on the tree branches follow the results of the present study.

Species boundaries recognized by 1.and 2. were cross-checked based on the results of ITS-based species delimitation analyses. When the species boundaries are supported by the majority (= more than four methods) of the seven species delimitation methods (UNITE SH at 3% threshold, UNITE SH at 1.5% threshold, VSEARCH 97% similarity, VSEARCH 98.5% similarity, ASAP, GMYC, and PTP) (Fig. 3), we regard the species as reasonable and carry out taxonomic treatments if necessary.

## ﻿Results

### ﻿Taxon sampling from TNS specimens

Forty-nine specimens in TNS were identified as candidates of *Erioscyphella* and morphologically identified as *E.abnormis*, *E.brasiliensis*, *E.sclerotii*, *Lachnumhainanense* W.Y. Zhuang & Zheng Wang, *L.mapirianum* (Pat. & Gaillard) M.P. Sharma, Lachnummapirianumvar.sinense Z.H. Yu, W.Y. Zhuang, Lachnumnovoguineensevar.yunnanicum W.Y. Zhuang, and *L.palmae* (Kanouse) Spooner (Table 1), together with six species of *Erioscyphella* described here as new ([*E.boninensis*, *E.insulae*, *E.otanii*, *E.papillaris*, *E.paralushanensis*, and *E.sasibrevispora*], Table 1).

### ﻿Phylogenetic analyses

The molecular phylogenetic analyses were based on 70 specimens selected from TNS (Table 1). The concatenated sequence matrix was composed of 2488 bp (sites 1–332 for ITS, 333–1108 for LSU, 1109–1828 for mtSSU, and 1829–2488 for RPB2). In the matrix, the following parts were treated as missing data: TNS-F-17245, 17249, and 81229 for mtSSU, and TNS-F-17567 for RPB2. The matrix was registered in TreeBase (http://purl.org/phylo/treebase/phylows/study/TB2:S28477).

Among the four ML trees based on each region, no conflicts were found in clades with support > 70% (Suppl. material 1: Fig. S1). Therefore, we considered these four regions to be combinable, and phylogenetic analyses were based on the concatenated sequence matrix. In the ML analysis, the best-scored tree generated did not reside on the phylogenetic terrace. In the MP analysis, 766 nucleotide substitution sites were detected, 601 of which were parsimony-informative. A total of 182,630 equally parsimonious trees were generated with tree length = 2,985 steps, consistency index (CI) = 0.38, retention index (RI) = 0.73, and rescaled consistency index (RC) = 0.28. In the BI analysis, when two runs reached 20 million generations and the first 10,000 trees (25%) of generated trees were excluded, ASDSF was observed to fall below 0.004 and ESS of all parameters was over 200. The first 10,000 trees were discarded as burn-in. A 50% majority rule consensus tree was constructed and BPP was calculated based on the remaining 30,000 trees.

As no topological contradictions occurred among the ML best-scored tree, MP 50% majority-rule consensus tree, and BI 50% majority-rule consensus tree, only ML tree was illustrated, and MLBS, MPBS, and BPP were plotted on its branches (Fig. 1).

Based on the phylogenetic analyses, 49 candidates of *Erioscyphella* formed a strongly supported clade (= Clade A, MLBP = 100%/MPBP = 100%/BPP = 1.00), apart from the clade of *Lachnum* sensu stricto (= *L.asiaticum* (Y. Otani) Raitv., *L.pudibundum* (Quél.) J. Schröt., *L.rachidicola* J.G. Han, Raitv. & H.D. Shin, and *L.virgineum* (Batsch) P. Karst.) [type of *Lachnum*]) (Fig. 1). Clade A and *Proliferodiscusalboviridis* formed a relatively strongly supported clade (Clade B, MLBP = 78%, MPBP = 82%, BPP = 1.00).

Within Clade A, each morphologically identified species and variety formed strongly supported monophyletic groups of their own (Fig. 1), and five strongly supported subclades were recognized (Clade I–V, Fig. 1). *Lachnummapirianum* (TNS-F-17545, 17249) and *E.insulae* (TNS-F-26500, 39720) did not belong to any subclade. Clade I was composed of *E.boninensis*, *E.paralushanensis*, *L.hainanense*, and L.mapirianumvar.sinense. Within Clade I, only *E.paralushanensis* occurred on bamboo sheaths, while others occurred on fallen leaves of broad-leaved trees. Clade II was composed only of *L.palmae*, which occurred on the palm petioles. Clade III was composed of *E.otanii* and *E.papillaris* occurring on bamboo leaves. Clade IV was composed of L.novoguineensevar.yunnanicum, and *E.sasibrevispora*, occurring on bamboo sheaths. Clade V was composed of *E.abnormis*, *E.brasiliensis*, and *E.sclerotii*, occurring on wood.

### ﻿Morphological characters within Clade A

Members of Clade A had totally and densely granulate, hyaline to brown, thin-walled hairs, fusiform to long filiform ascospores, ectal excipulum composed of *textura prismatica* to *textura angularis*, asci lacking croziers at the bases, and smooth walled ectal excipulum cells. Exceptionally, *E.sasibrevispora*, *L.hainanense* (Hosoya et al. 2013), and L.novoguineensevar.yunnanicum W.Y. Zhuang had croziers and *E.boninensis* had granulated ectal excipulum.

Moreover, hairs of Clade A lacked crystals, but were equipped with apical amorphous materials and/or resinous materials. In the present study, “crystals” refers to amber colored materials that positioned near the hair apices and were regular-shaped (e.g. tetrahedral materials, masses of needle-like materials, or cross-shaped materials), described by Raitviir (2002), Suková (2005) or Tochihara and Hosoya (2019). “Resinous materials” refers to colored, refractive, irregular-shaped materials attached on any parts of hairs, described by Spooner (1987). Crystals and resinous materials are easily detatched from hairs and broken into fragments in the squash mount. “Apical amorphous materials” is termed uniquely in this study, and refers to hyaline to brown, refractive, irregular-shaped materials positioned outside the hair apices. They are usually small and inconspicuous cap-like shaped, and conspicuously globular in some species. Apical amorphous materials do not grow to big masses and are not easily detached from hairs in the squash mount.

In Clade A, members except for *E.boninensis*, *E.sasibrevispora* and L.novoguineensevar.yunnanicum had apical amorphous materials, and *E.boninensis*, *E.paralushanensis*, and *L.palmae* complex also had resinous materials (see figures of described species and Suppl. material 1: Fig. S2).

### ﻿ITS-based species delimitation analyses

In UNITE v8.3, 87 ITS sequences were clustered into 23 SHs at 3% and 26 SHs at 1.5% threshold values (Table 2, Fig. 3). The UNITE SH code for each SH is presented in Table 2. In OTU clustering using VSEARCH, 87 ITS sequences were clustered into 25 SHs at 97% similarity and 28 SHs at 98.5% similarity (Table 2, Fig. 3). VSEARCH SH codes (allocated in this study uniquely; VSH97_1 to VSH97_25, VSH985_1 to VSH985_28) are shown in Table 2.

**Table 2.** ITS sequences analyzed by the species delimitation analyses.

ITS sequence GenBank/UNITE accession no.	TNS-F specimen no.	Reference (initial appearance)	Taxon name (ultimately allocated in this study)	UNITE taxon name	INSDC taxon name	Country	Host plants and parts	UNITE SH code (DOI) at 3% threshold	UNITE SH code (DOI) at 1.5% threshold	VSEARCH SH at 97% similarity	VSEARCH SH at 98.5% similarity
AB267634		Miyoshi et al. (2007)	*E.abnormis*	*Lachnumabnorme*	*Lachnumabnorme*	JAPAN, Ehime	twig of *Citrusjunos*	SH1155612.08FU	SH1522994.08FU	VSH97_1	VSH985_2
AB267636 (duplicate; AB267635)		Miyoshi et al. (2007)	*E.abnormis*	*Lachnumabnorme*	*Lachnumabnorme*	JAPAN, Ehime	twig of *Citrusjunos*	SH1155612.08FU	SH1522994.08FU	VSH97_1	VSH985_2
AB267641 (duplicate; AB267639, AB267640)		Miyoshi et al. (2007)	*E.abnormis*	*Lachnumabnorme*	*Lachnumabnorme*	JAPAN, Tokushima	twig of *Citrusjunos*	SH1155612.08FU	SH1522994.08FU	VSH97_1	VSH985_2
AB267642		Miyoshi et al. (2007)	*E.abnormis*	*Lachnumabnorme*	*Lachnumabnorme*	JAPAN, Tokushima	twig of *Citrusjunos*	SH1155612.08FU	SH1522994.08FU	VSH97_1	VSH985_2
JF937578		Zhao and Zhuang (2011)	*E.abnormis*	*Lachnumabnorme*	*Lachnumabnorme*	CHINA	(unspecified)	SH1155612.08FU	SH1522994.08FU	VSH97_1	VSH985_2
JN033395		Han et al. (2014)	*E.abnormis*	*Lachnumabnorme*	*Lachnumabnorme*	KOREA	Wood	SH1155612.08FU	SH1522994.08FU	VSH97_1	VSH985_2
UDB0779067/LC669455	46416	this study	*E.abnormis*	-	-	TAIWAN, Taipei	wood of unidentified tree	SH1155612.08FU	SH1522994.08FU	VSH97_1	VSH985_2
UDB0779074/LC669462	61773	this study	*E.abnormis*	-	-	JAPAN, Kanagawa, Yokohama	twig of unidentified tree	SH1155612.08FU	SH1522994.08FU	VSH97_1	VSH985_2
MK584950		Ekanayaka et al. (2019)	*E.abnormis*	*E.abnormis*	*E.abnormis*	CHINA, Yunnan	(unspecified)	SH1155612.08FU	†SH1522994.08FU	VSH97_1	VSH985_2
AB267637		Miyoshi et al. (2007)	*E.abnormis*	*Lachnumabnorme*	*Lachnumabnorme*	JAPAN, Nara	Twig	SH1155612.08FU	SH1523013.08FU	VSH97_2	VSH985_1
AB267638		Miyoshi et al. (2007)	*E.abnormis*	*Lachnumabnorme*	*Lachnumabnorme*	JAPAN, Shizuoka	Twig	SH1155612.08FU	SH1523013.08FU	VSH97_2	VSH985_1
AB481249	16582	Hosoya et al. (2010)	*E.abnormis*	*Lachnumabnorme*	*Lachnumabnorme*	JAPAN, Kanagawa, Yamakita	wood of unidentified tree	SH1155612.08FU	SH1523013.08FU	VSH97_1	VSH985_1
AB705234	16609	Zhao et al. (2012)	*E.abnormis*	*Lachnumabnorme*	*Lachnumabnorme*	JAPAN, Kanagawa, Yamakita	wood of *Cephalotaxusharringtonia*	SH1155612.08FU	SH1523013.08FU	VSH97_1	VSH985_1
LC424837	80478	this study	*E.abnormis*	-	-	JAPAN, Shizuoka, Oyama	twig of unidentified tree	SH1155612.08FU	SH1523013.08FU	VSH97_2	VSH985_3
MG712307		unpublished	*E.abnormis*	*Lachnumabnorme*	*Lachnumabnorme*	CHINA	(unspecified)	SH1155612.08FU	SH1523013.08FU	VSH97_2	VSH985_3
MK282241		unpublished	*E.abnormis*	*Lachnumabnorme*	*Lachnumabnorme*	(unspecified)	(unspecified)	SH1155612.08FU	SH1523013.08FU	VSH97_2	VSH985_1
MK584957		Ekanayaka et al. (2019)	*E.abnormis*	*E.aseptata*	*E.aseptata*	THAILAND, Chiang Rai	(unspecified)	SH1155612.08FU	SH1523013.08FU	VSH97_2	VSH985_1
MN082536		unpublished	*E.abnormis*	*Lachnumabnorme*	*Lachnumabnorme*	(unspecified)	(unspecified)	SH1155612.08FU	SH1523013.08FU	VSH97_1	VSH985_1
MT995055		unpublished	*E.abnormis* (misregistered?)	*Chapsapatens*	*Chapsapatens*	(unspecified)	(unspecified)	SH1155612.08FU	SH1523013.08FU	VSH97_1	VSH985_1
MW007918		unpublished	*E.abnormis* (misregistered?)	*Chapsapatens*	*Chapsapatens*	(unspecified)	(unspecified)	SH1155612.08FU	SH1523013.08FU	VSH97_2	VSH985_3
UDB0779051/LC669439	16556	this study	*E.abnormis*	-	-	JAPAN, Oita, Kokonoe	wood of unidentified tree	SH1155612.08FU	SH1523013.08FU	VSH97_2	VSH985_1
UDB0779053/LC669441	16606	this study	*E.abnormis*	-	-	JAPAN, Kanagawa, Yamakita	wood of unidentified tree	SH1155612.08FU	SH1523013.08FU	VSH97_2	VSH985_3
UDB0779054/LC669442	16639	this study	*E.abnormis*	-	-	JAPAN, Ibaraki, Tsukuba Botanical Garden	twig of unidentified tree	SH1155612.08FU	SH1523013.08FU	VSH97_2	VSH985_3
UDB0779057/LC669445	25579	this study	*E.abnormis*	-	-	JAPAN, Tokyo, Hongo	twig of unidentified tree	SH1155612.08FU	SH1523013.08FU	VSH97_2	VSH985_3
UDB0779062/LC669450	32163	this study	*E.abnormis*	-	-	JAPAN, Kanagawa, Odawara	twig of unidentified tree	SH1155612.08FU	SH1523013.08FU	VSH97_2	VSH985_3
UDB0779069/LC669457	38452	this study	*E.abnormis*	-	-	JAPAN, Gunma, Naganohara	twig of unidentified tree	SH1155612.08FU	SH1523013.08FU	VSH97_1	VSH985_1
UDB0779072/LC669460	61931	this study	*E.abnormis*	-	-	JAPAN, Kanagawa, Zushi	twig of unidentified tree	SH1155612.08FU	SH1523013.08FU	VSH97_2	VSH985_3
UDB0779086/LC669474	46841	this study	*E.abnormis*	-	-	JAPAN, Gifu, Gujo	twig of unidentified tree	SH1155612.08FU	SH1523013.08FU	VSH97_1	VSH985_1
AB481250	16617	Hosoya et al. (2010)	*E.abnormis*	*Lachnumabnorme*	*Lachnumabnorme*	JAPAN, Kanagawa, Yamakita	twig of unidentified tree	‡SH1155612.08FU	‡SH1523013.08FU	VSH97_1	VSH985_1
UDB0779055/LC669443	16837	this study	*E.sinensis* (←Lachnummapirianumvar.sinense)	-	-	JAPAN, Ibaraki, Tsukuba Botanical Garden	leaf of unidentified broad-leaved tree	SH1155682.08FU	SH1523107.08FU	VSH97_4	VSH985_5
AB481280	16838	Hosoya et al. (2010)	*E.sinensis* (←Lachnummapirianumvar.sinense)	*Lachnum* sp.	*Lachnum* (*Lachnum* sp. FC-2355)	JAPAN, Ibaraki, Tsukuba Botanical Garden	leaf of unidentified broad-leaved tree	SH1155682.08FU	SH1523107.08FU	VSH97_4	VSH985_5
AB481281	16841	Hosoya et al. (2010)	*E.sinensis* (←Lachnummapirianumvar.sinense)	*Lachnum* sp.	*Lachnum* (*Lachnum* sp. FC-2358)	JAPAN, Ibaraki, Mt. Tsukuba	leaf of unidentified broad-leaved tree	SH1155682.08FU	SH1523107.08FU	VSH97_4	VSH985_5
UDB0779061/LC669449	32161	this study	*E.sinensis* (←Lachnummapirianumvar.sinense)	-	-	JAPAN, Kanagawa, Odawara	leaf of *Quercusmyrsinifolia*	SH1155682.08FU	SH1523107.08FU	VSH97_4	VSH985_5
UDB0779083/LC669471	80354	this study	*E.sinensis* (←Lachnummapirianumvar.sinense)	-	-	JAPAN, Kanagawa, Manazuru	leaf of *Castanopsissieboldii*	†SH1155682.08FU	†SH1523107.08FU	VSH97_4	VSH985_5
UDB023346		unpublished	*E.curvispora*	*E.curvispora*	-	MONTENEGRO, Žijevo Mountains	needle of *Pinusheldreichii*	SH1155703.08FU	SH1523136.08FU	VSH97_12	VSH985_14
MH190414		Perić and Baral (2014)	*E.curvispora*	*E.curvispora*	*E.curvispora*	MONTENEGRO, Žijevo Mountains	needle of *Pinusheldreichii*	†SH1155703.08FU	†SH1523136.08FU	VSH97_12	VSH985_14
JF937580		Zhao and Zhuang (2011)	*E.brasiliensis*	*Lachnumbrasiliense*	*Lachnumbrasiliense*	CHINA	(unspecified)	SH1155705.08FU	SH1523142.08FU	VSH97_6	VSH985_7
MK584953		Ekanayaka et al. (2019)	*E.brasiliensis*	*E.brasiliensis*	*E.brasiliensis*	(unspecified)	(unspecified)	SH1155705.08FU	SH1523142.08FU	VSH97_6	VSH985_7
MK584967		Ekanayaka et al. (2019)	*E.brasiliensis*	*E.brasiliensis*	*E.brasiliensis*	THAILAND, Chiang Rai	(unspecified)	SH1155705.08FU	SH1523142.08FU	VSH97_6	VSH985_7
UDB0779068/LC669456	46419	this study	*E.brasiliensis*	-	-	TAIWAN, Taipei	wood of unidentified tree	SH1155705.08FU	SH1523142.08FU	VSH97_6	VSH985_7
JF937579		Zhao and Zhuang (2011)	*E.brasiliensis*	*Lachnumbrasiliense*	*Lachnumbrasiliense*	CHINA	(unspecified)	†SH1155705.08FU	†SH1523142.08FU	VSH97_6	VSH985_7
KX501132		Tello and Baral (2016)	*E.lunata*	*E.lunata*	*E.lunata*	SPAIN, Andalucía	needle of Pinusnigrasubsp.nigra	†SH1155760.08FU	†SH1523257.08FU	VSH97_18	VSH985_19
JX984680		unpublished	*E.hainanensis* (←*Lachnumhainanense*)	Hyaloscyphaceae	Fungi (uncultured fungus)	KOREA, Seoul	(Total suspended particulate matter (TSP) in urban air during non-Asian dust days)	SH1155844.08FU	SH1523423.08FU	VSH97_3	VSH985_4
UDB0779064/LC669452	35049	this study	*E.hainanensis* (←*Lachnumhainanense*)	-	-	JAPAN, Niigata, Minamiuonuma	leaf of *Quercusglauca*	SH1155844.08FU	SH1523423.08FU	VSH97_3	VSH985_4
UDB0779065/LC669453	35056	this study	*E.hainanensis* (←*Lachnumhainanense*)	-	-	JAPAN, Niigata, Minamiuonuma	leaf of *Quercusserrata*	SH1155844.08FU	SH1523423.08FU	VSH97_3	VSH985_4
UDB0779073/LC669461	61941	this study	*E.hainanensis* (←*Lachnumhainanense*)	-	-	JAPAN, Kanagawa, Kamakura	leaf of *Quercusglauca*	SH1155844.08FU	SH1523423.08FU	VSH97_3	VSH985_4
UDB0779076/LC669464	65722	this study	*E.hainanensis* (←*Lachnumhainanense*)	-	-	JAPAN, Gunma, Midori	leaf of Quercusserratasubsp.mongolicoides	SH1155844.08FU	SH1523423.08FU	VSH97_3	VSH985_4
MK282242		unpublished	*E.hainanensis* (←*Lachnumhainanense*)	*Lachnum* sp.	*Lachnumalbidulum*	KOREA	(unspecified)	SH1155844.08FU	†SH1523423.08FU	VSH97_3	VSH985_4
UDB0779077/LC669465	80356	this study	*E.hainanensis* (←*Lachnumhainanense*)	-	-	JAPAN, Kanagawa, Hiratsuka	leaf of *Quercusglauca*	SH1155844.08FU	SH3597461.08FU	VSH97_3	VSH985_9
UDB0779078/LC669466	80371	this study	*E.hainanensis* (←*Lachnumhainanense*)	-	-	JAPAN, Kanagawa, Hiratsuka	leaf of *Castanopsissieboldii*	SH1155844.08FU	SH3597461.08FU	VSH97_3	VSH985_9
UDB0779071/LC669459	61775	this study	*E.hainanensis* (←*Lachnumhainanense*)	-	-	JAPAN, Kanagawa, Hiratsuka	leaf of *Quercusmyrsinifolia*	†SH1155844.08FU	†SH3597461.08FU	VSH97_3	VSH985_9
UDB0779050/LC669438	26492	this study	*E.sclerotii*	-	-	JAPAN, Tokyo, Hahajima Island	wood of unidentified tree	SH1155848.08FU	SH1523429.08FU	VSH97_5	VSH985_6
JF937584		Zhao and Zhuang (2011)	*E.sclerotii*	*Lachnumsclerotii*	*Lachnumsclerotii*	CHINA	(unspecified)	SH1155848.08FU	SH1523429.08FU	VSH97_5	VSH985_6
MK584951		Ekanayaka et al. (2019)	*E.sclerotii*	*E.sclerotii*	*E.sclerotii*	THAILAND, Chiang Rai	(unspecified)	SH1155848.08FU	SH1523429.08FU	VSH97_5	VSH985_6
UDB0779070/LC669458	38480	this study	*E.sclerotii*	-	-	TAIWAN, Wulai	twig of unidentified tree	SH1155848.08FU	SH1523429.08FU	VSH97_5	VSH985_6
MK584969		Ekanayaka et al. (2019)	*E.sclerotii*	*E.sclerotii*	*E.sclerotii*	THAILAND, Chiang Rai	(unspecified)	†SH1155848.08FU	†SH1523429.08FU	VSH97_5	VSH985_6
AB481271	16642	Hosoya et al. (2010)	Lachnumnovoguineensevar.yunnanicum	*Lachnum* sp.	*Lachnum* sp. (*Lachnum* sp. FC-2211)	JAPAN, Ibaraki, Mt. Tsukuba	culm of unidentified bamboo	SH1236904.08FU	SH1648536.08FU	VSH97_10	VSH985_12
AB481270	16442	Hosoya et al. (2010)	Lachnumnovoguineensevar.yunnanicum	*Lachnum* sp.	*Lachnum* sp. (*Lachnum* sp. FC-2117)	JAPAN, Nagano, Ueda, Sugadaira Montane Research Center	culm of unidentified bamboo	†SH1236904.08FU	†SH1648536.08FU	VSH97_10	VSH985_12
MK584965		Ekanayaka et al. (2019)	*E.alba*	*E.alba*	*E.alba*	THAILAND, Chiang Mai	(unspecified)	†SH2596405.08FU	†SH2712425.08FU	VSH97_22	VSH985_25
AB267647		Miyoshi et al. (2007)	*Lachnumpalmae* sensu lato	*Lachnumpalmae*	*Lachnumpalmae*	JAPAN, Oita	leaf of *Livistonachinensis*	SH1149764.08FU	SH1515235.08FU	VSH97_7	VSH985_8
LC425039 (duplicate; UDB0779046)	13500	Johnston et al. (2019)	*Lachnumpalmae* sensu lato	*Lachnumpalmae*	*Lachnumpalmae*	JAPAN, Kagoshima, Yakushima Island	leaf of Livistonachinensisvar.subglobosa	SH1149764.08FU	SH1515235.08FU	VSH97_7	VSH985_8
UDB0779066/LC669454	39729	this study	*Lachnumpalmae* sensu lato	-	-	JAPAN, Okinawa, Iriomote Island	leaf of Livistonachinensisvar.subglobosa	SH1149764.08FU	SH1515235.08FU	VSH97_7	VSH985_8
MG283320		Zhao et al. (2018)	*Lachnumpalmae* sensu lato	*Lachnumpalmae*	*Lachnumpalmae*	CHINA, Linzhou	root of *Przewalskiatangutica* (endophyte)	†SH1149764.08FU	†SH1515235.08FU	VSH97_7	VSH985_8
UDB0779089/LC669477	17567	this study	*Lachnumpalmae* sensu lato	-	-	NEW ZEALAND	leaf of unidentified palm	SH2594271.08FU	SH2709065.08FU	VSH97_15	VSH985_16
MH921862		unpublished	*Lachnumpalmae* sensu lato	*Lachnumpalmae*	*Lachnumpalmae*	NEW ZEALAND	unidentified part of *Rhopalostylissapida*	†SH2594271.08FU	†SH2709065.08FU	VSH97_15	VSH985_16
UDB0779052/LC669440	24588	this study	*Lachnumpalmae* sensu lato	-	-	JAPAN, Kagoshima, Amami-Oshima	leaf of Livistonachinensisvar.subglobosa	SH3569651.08FU	SH3597456.08FU	VSH97_9	VSH985_17
UDB0779047/LC669435	11197	this study	*Lachnumpalmae* sensu lato	-	-	JAPAN, Shizuoka, Shimoda	leaf of Livistonachinensisvar.subglobosa	†SH3569651.08FU	†SH3597456.08FU	VSH97_9	VSH985_17
UDB0779048/LC669436	26161	this study	*Lachnumpalmae* sensu lato	-	-	JAPAN, Tokyo, Chichijima Island	leaf of *Livistonaboninensis*	SH3569651.08FU	SH3597457.08FU	VSH97_9	VSH985_11
UDB0779058/LC669446	26172	this study	*Lachnumpalmae* sensu lato	-	-	JAPAN, Tokyo, Kita-Iwojima Island	leaf of Livistonachinensisvar.subglobosa	SH3569651.08FU	SH3597457.08FU	VSH97_16	VSH985_11
UDB0779059/LC669447	26185	this study	*Lachnumpalmae* sensu lato	-	-	JAPAN, Tokyo, Kita-Iwojima Island	leaf of Livistonachinensisvar.subglobosa	SH3569651.08FU	†SH3597457.08FU	VSH97_16	VSH985_11
UDB0779056/LC669444	24600	this study	*Lachnumpalmae* sensu lato	-	-	JAPAN, Kagoshima, Amami-Oshima	leaf of Livistonachinensisvar.subglobosa	†SH3569653.08FU	†SH3597459.08FU	VSH97_25	VSH985_28
U58640		Cantrell and Hanlin (1997)	*E.euterpes*	*Lachnumeuterpes*	*Lachnumeuterpes*	PUERTO RICO	(unspecified)	†SH1236906.08FU	†SH1648538.08FU	VSH97_21	VSH985_24
KT384413		Ekanayaka et al. (2019)	*E.fusiformis*	*Lachnumfusiforme*	*Lachnumfusiforme*	THAILAND	dead stems	‡SH1236907.08FU	‡SH1648539.08FU	VSH97_11	VSH985_13
MK584948		Ekanayaka et al. (2019)	*E.fusiformis*	*Lachnumfusiforme*	*Lachnumfusiforme*	CHINA	dead stems	SH1236907.08FU	SH1648539.08FU	VSH97_11	VSH985_13
UDB0779049/LC669437	26520	this study	*E.boninensis*	-	-	JAPAN, Tokyo, Hahajima Island	wood of unidentified tree	†SH3569652.08FU	†SH3597458.08FU	VSH97_20	VSH985_21
UDB0779060/LC669448	26500	this study	*E.insulae*	-	-	JAPAN, Tokyo, Hahajima Island	wood of unidentified tree	SH3569654.08FU	SH3597460.08FU	VSH97_14	VSH985_15
UDB0779063/LC669451	39720	this study	*E.insulae*	-	-	JAPAN, Okinawa, Iriomote Island	bark of unidentified tree	†SH3569654.08FU	†SH3597460.08FU	VSH97_14	VSH985_15
UDB0779075/LC669463	61920	this study	*E.paralushanensis*	-	-	JAPAN, Shizuoka, Atami	culm of *Pleioblastusargenteostriatus*	†SH3569655.08FU	†SH3597462.08FU	VSH97_19	VSH985_20
AF505515			*E.lushanensis*	*Lachnumlushanense*	*Lachnumlushanense*	(unspecified)	(unspecified)	†SH1155706.08FU	†SH1523143.08FU	VSH97_8	VSH985_10
JF937582		Zhao and Zhuang (2011)	*E.lushanensis*	*Lachnumlushanense*	*Lachnumlushanense*	CHINA	(unspecified)	SH1155706.08FU	SH1523143.08FU	VSH97_8	VSH985_10
MG434782		unpublished	*E.lushanensis*	*Erioscyphella* sp.	*E.lushanensis*	INDIA, Tangmarg	root tips of *Pinuswallichiana* (ectomycorrhiza)	(unassigned)	(unassigned)	VSH97_8	VSH985_10
UDB0779081/LC669469	81272	this study	*E.papillaris*	-	-	JAPAN, Gunma, Minakami	leaf of unidentified bamboo	†SH3569656.08FU	†SH3597463.08FU	VSH97_23	VSH985_26
UDB0779084/LC669472	81401	this study	*E.sasibrevispora*	-	-	JAPAN, Hokkaido, Tomakomai	culm of *Sasanipponica*	SH3569657.08FU	SH3597464.08FU	VSH97_13	VSH985_23
UDB0779082/LC669470	80399	this study	*E.sasibrevispora*	-	-	JAPAN, Gunma, Higashi-Agatsuma	sheath of *Sasaveitchii*	†SH3569657.08FU	†SH3597464.08FU	VSH97_13	VSH985_22
UDB0779085/LC669473	81472	this study	*E.otanii*	-	-	JAPAN, Hokkaido, Horonobe, Teshio Experimental Forest, Hokkaido University	leaf of *Sasasenanensis*	†SH3569658.08FU	†SH3597465.08FU	VSH97_24	VSH985_27
UDB0779087/LC669475	17245	this study	*Lachnummapirianum*	-	-	MALAYSIA, Gerik	leaf of unidentified tree	†SH3569659.08FU	†SH3597466.08FU	VSH97_17	VSH985_18
UDB0779088/LC669476	17249	this study	*Lachnummapirianum*	-	-	MALAYSIA, Gerik	leaf of unidentified tree	SH3569659.08FU	SH3597466.08FU	VSH97_17	VSH985_18

† Representative sequence of each SH ‡ Reference sequence of each SH

The extracted and aligned ITS sequences were composed of three partitions, ITS1 (162 bp), 5.8S (157 bp), and ITS2 (142 bp). The concatenated ITS sequence matrix was registered in TreeBase (http://purl.org/phylo/treebase/phylows/study/TB2:S28473). In the ASAP analysis, the concatenated dataset of these partitions (461 bp) was input, and 87 ITS sequences were clustered into 18 SHs with the lowest asap-score, reflecting better partitioning (Suppl. material 1: Fig. S3). In the GMYC analysis, 29 SHs were delimited (Suppl. material 1: Fig. S4). The ultrametric tree constructed for the GMYC analysis is available in TreeBase (http://purl.org/phylo/treebase/phylows/study/TB2:S28473). For the PTP analyses, an ML best-scored tree was constructed (Suppl. material 1: Fig. S5). PTP analyses delimited 23 SHs in the Bayesian support and 26 SHs in the ML support (Suppl. material 1 Fig. S6), and the former was adopted.

Comparing the number of SHs generated by different clustering methods and applied thresholds, 18 SHs by ASAP, and 23 SHs by UNITE SH at 3% threshold represented the lowest SH numbers (Fig. 3; Table 2). The ASAP results were too rough to delimit the boundaries of *E.abnormis*, *E.boninensis*, *E.brasiliensis*, *E.curvispora*, and *E.sclerotii*. SH-classification recognized by UNITE SH at 3% threshold mostly corresponded to taxon names originally assigned to sequences.

Comparing the results of seven species delimitation methods (UNITE SH at 3% threshold, UNITE SH at 1.5% threshold, VSEARCH 97% similarity, VSEARCH 98.5% similarity, ASAP, GMYC, and PTP), sequences labeled as *E.alba*, *E.brasiliensis*, *E.curvispora*, *E.euterpes*, *E.fusiformis*, *E.lunata*, *E.sclerotii*, *L.mapirianum*, L.mapirianumvar.sinense, L.novoguineensevar.yunnanica, and six new species candidates were distinguished as separate clusters by more than four delimitation methods (Fig. 3). These species clusters did not contradict with morphological/ecological and phylogenetic relationships (Fig. 1). Seven sequences labeled as *L.hainanense* were clustered into one SH by four species delimitation analyses, and part of the SHs included a sequence labeled as *Lachnumalbidulum* (Fig. 3).

*Erioscyphellaabnormis*, *E.aseptate*, and *L.palmae* did not form separate clusters supported by majority of four species delimitation analyses (Fig. 3). Sequences labeled as *E.abnormis* were clustered into one to four SHs, and some SHs included sequences labeled as *Chapsapatens* (Nyl.) Frisch, *E.aseptata*, *E.brasiliensis*, and *E.sclerotii* (Fig. 3). Twelve sequences labeled as *L.palmae* were clustered into four to six SHs (Fig. 3).

## ﻿Discussion

### ﻿Generic delimitation and generic concept of *Erioscyphella*

**Figure 4.**
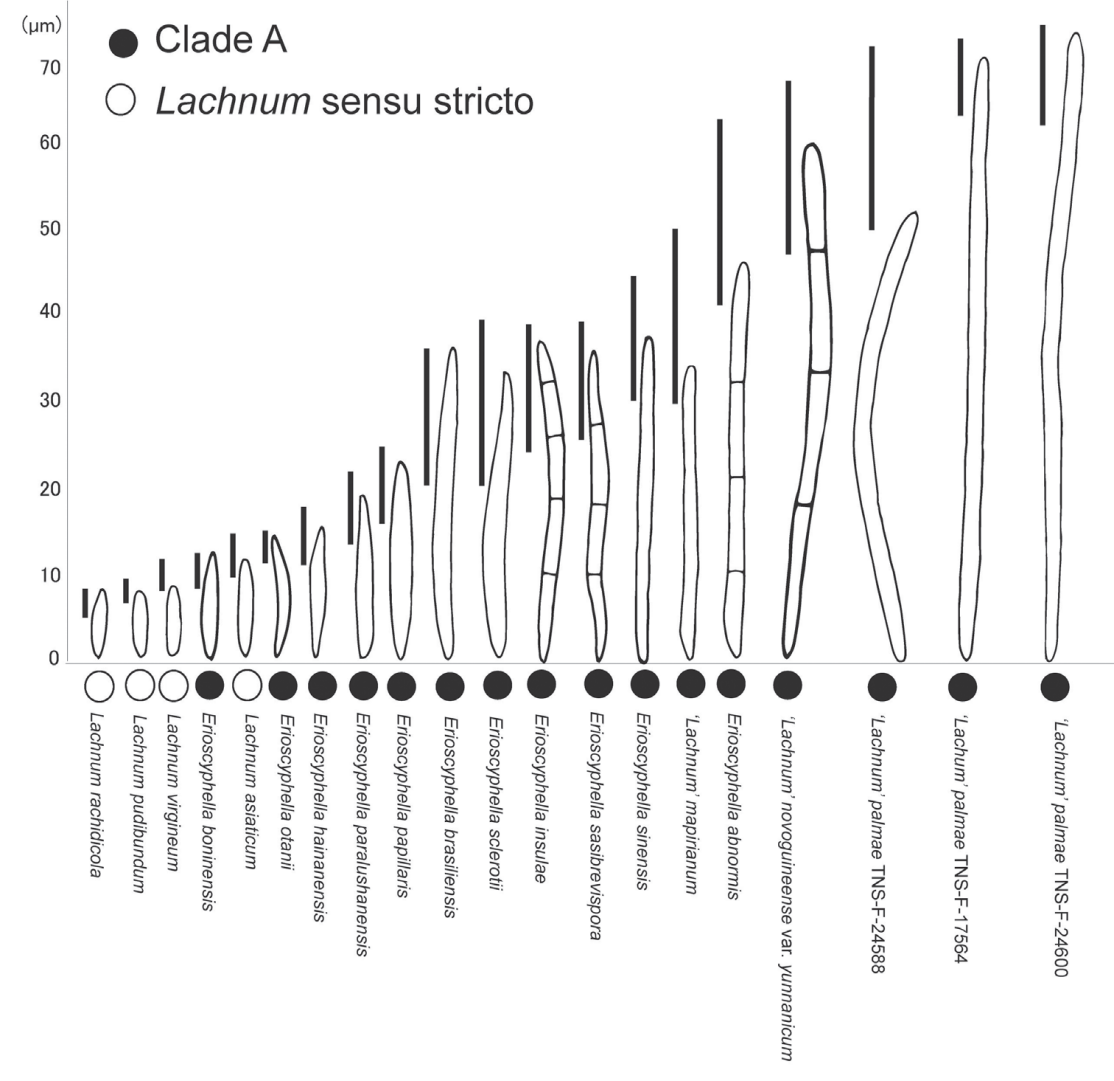
Comparison of ascospores of Clade A (= *Erioscyphella*) and the clade of *Lachnum* sensu stricto in Fig. 1. Subclade numbers for members of Clade A in Fig. 1 are shown in parentheses. Bars show variation of ascospore length within each species.

We accepted Clade A as a monophyletic unit for *Erioscyphella* which is supported by morphology. Although Clade B comprised Clade A together with *P.alboviridis*, Clade B should not be regarded as a genus delimitation of *Erioscyphella*, because *Proliferodiscus* differs from members of Clade A in having apothecia proliferating from the margins continuously and thick-walled and coarsely warted hairs (Haines and Dumont 1983; Spooner 1987). All members of Clade A are distinguishable from the other lachnacenous genera. In contrast to *Erioscyphella*, *Albotricha* and *Dasyscyphella* are distinguished by hair apices with no granulation (Hosoya et al. 2010), *Brunnipila*, *Capitotricha*, and *Incrucipulum* by hair-crystals (Baral and Krieglsteiner 1985; Tochihara and Hosoya 2019), and *Lachnellula* by ectal excipulum composed of *textura globose* to *textura oblita* (Dharne 1965). Typical members of Clade A can be easily segregated from *Neodasyscypha*, because the characteristic features of *Neodasyscypha*, such as dark-brown hairs, ectal-excipulum structure, and ellipsoid to fusoid ascospores < 10 µm long (Spooner 1987), are rare in Clade A. Among members of Clade A and *Lachnum* sensu stricto, the shape and length of ascospores were continuous (Fig. 4), as indicated by Haines and Dumont (1984). However, ascospores longer than 15–20 µm were restricted to Clade A (Fig. 4). Moreover, most members of Clade A have hairs with apical amorphous materials, which are not seen in *Lachnum* sensu stricto. Members of Clade A usually also have hairs not swelling at the apices and distantly septate, as Perić and Baral (2014) pointed out for three tropical members, while members of *Lachnum* have swelling apices. The combination of such characters allows us to differentiate typical members of *Erioscyphella* from *Lachnum*.

In summary, *Erioscyphella* is still difficult to define solely based on morphology because of multiple exceptional characters continuous to other genera, but its typical members could be recognizable mainly by the hair structures and ascospore length. Based on members of Clade A, *Erioscyphella* is tentatively described as follows: apothecia occurring on dead hardwood leaves, rotten wood, bamboo sheaths, bamboo leaves or palm leaves; asci mostly arising from simple septa, but occasionally from croziers; ascospores fusiform to long needle-shaped, aseptate to multi-septate; paraphyses filiform to narrowly lanceolate, shortly exceeding the asci, but rarely lanceolate and long exceeding the asci; hairs straight or irregularly curved, usually not swollen at the apices, thin-walled, hyaline, but sometimes brown, totally and densely granulated, usually distantly septate, without needle-like or three-dimensional shaped crystals but mostly equipped with hyaline to brown apical amorphous materials, and/or resinous materials at any part of hairs; walls of ectal excipulum cells smooth but granulate in one species.

Perić and Baral (2014) pointed out that “yellow hymenium derived from carotenoid” is one of the common characters of *Erioscyphella*. This feature was not discussed in this study because some specimens were not observed when fresh; the hymenium color is variable (usually white hymenium becomes yellow) between fresh and dried states in lachnaceous species.

### ﻿Host selectivity of *Erioscyphella*

In *Erioscyphella*, the tendency of selectivity of species to host plants or parts occurs across the genus. Each subclade within *Erioscyphella* (Clade I–V) generally shared tendencies toward host selectivity as follows: Clade I on leaves of broad-leaved trees, except for *E.paralushanensis* occurring on bamboo sheaths, Clade II on palm leaves, Clade III on bamboo leaves, Clade IV on bamboo sheaths, and Clade V on rotten wood (Fig. 1). The results showed that selectivity to host plants, and parts of *Erioscyphella*, was acquired as apomorphic characters during speciation.

### ﻿Is *Erioscyphella* limited to ‘tropical’ zones?

*Erioscyphella* (long-spored *Lachnum*) has long been known as the tropical genus in Lachnaceae (Dennis 1954; Spooner 1987; Guatimosim et al. 2016). Most long-spored species were described from tropical areas of Latin America (Dennis 1954) and tropical to temperate areas of Australasia (Spooner 1987). However, the new species or new combinations proposed in this study were reported from Japan in subtropical areas (*E.boninensis* and *E.insulae*), temperate area (*E.hainanensis*, *E.palalushanensis*, and *E.sinensis*) and cool-temperate to subarctic areas (*E.otanii*, *E.papillaris*, and *E.sasibrevispora*), showing that *Erioscyphella* is not limited to tropical zones, but is also distributed in temperate to subarctic zones in the northern hemisphere.

### ﻿Ascal iodine reactions seen in *E.papillaris*

Iodine reactions of the ascus apical apparatus have been classified into several types (inamyloid, hemiamyloid [Type RB and RR, and euamyloid Type BB]) (Baral 2009), and the reaction ‘MLZ- without KOH pretreatment and MLZ+ with KOH pretreatment’, observed in *E.papillaris* (Fig. 11E1 and Fig. 11E2) has been restricted to the type of hemiamyloid. However, the apical apparatus of *E.papillaris* showed a dark blue reaction in IKI without KOH pretreatment (Fig. 11E3), while the hemiamyloid apparatus usually shows a red reaction under these conditions. The hemiamyloid ascal apparatus could show IKI-blue without KOH pretreatment due to long storage in the herbarium (Baral 2009), but this is not applicable for the material of *E.papillaris*, which has been maintained for only two years in herbarium until observed. Therefore, we assessed the iodine reaction of *E.papillaris* as a new type, and color reactions with various solutions of the species should be further examined using new materials, because there are few apothecia in the type specimen.

### ﻿Species-level taxonomic treatment of *Erioscyphella*

In this study, we carried out taxonomic treatment for species which were distinguished by morphology/ecology and phylogenetic analyses, and formed single clusters in species delimitation analyses. Based on this criteria, six undescribed species of *Erioscyphella* have been proposed as new species of *Erioscyphella* [*E.boninensis*, *E.insulae*, *E.otanii*, *E.papillaris*, *E.paralushanensis*, and *E.sasibrevispora*], and *Lachnumhainanense* and L.mapirianumvar.sinense have been proposed as new members of *Erioscyphella*. Interpretation of species boundaries of *L.hainanense* was discussed in the taxonomy chapter. For new species and new combinations, Japanese names were also denominated for wider use of Japanese mycologists or amateurs.

In the phylogenetic analyses, Malaysian materials of *L.mapirianum* (TNS-F-17245, 17249) and Japanese materials of L.novoguineensevar.yunnanicum (TNS-F-16442, 16642) were also found to be members of *Erioscyphella* (Fig. 1). However, we hesitate to transfer the two species into *Erioscyphella*, as we cannot guarantee the identification accuracy of the materials, because of inadequate type information of the two species.

Taxonomic assessments of *E.abnormis*, *L.aseptate*, and *L.palmae*, which were not accepted as independent species in species delimitation analyses, are discussed below.

### ﻿Taxonomy of *E.abnormis* and its related species

In the species delimitation analyses, sequences labeled as *E.abnormis* formed a single SH at UNITE SH 3% threshold (DOI: SH1155612.08FU) and divided into two to four SHs at UNITE SH 1.5% threshold, VSEARCH, and GMYC (Fig. 3).

In ASAP, sequences labeled as *E.abnormis* belong to a single SH, but the SH also contained sequences labeled as *Chapsapatens*, *E.aseptata*, *E.brasiliensis*, *E.curvispora*, and *E.sclerotii* (Fig. 3). However, the phylogenetic analyses revealed that *E.brasiliensis*, and *E.sclerotii* are separate from the clade of *E.abnormis* (Fig. 1), suggesting that the two species are different from *E.abnormis*. Although *E.curvispora* was not included in the phylogenetic analyses (Fig. 1), the apparent morphological and ecological differentiation (Perić and Baral 2014) and low similarity of ITS (< 97%) with members of *E.abnormis* (Fig. 3) suggest that *E.curvispora* is different from *E.abnormis*.

*Erioscyphellaaseptata* was originally described in Thailand and characterized by having aseptate ascospores, unlike *E.abnormis* or *E.sclerotii* with septate ascospores (Ekanayaka et al. 2019). However, the species delimitation analyses in this study suggested the difficulty of delimiting *E.aseptata* (MK584957) from *E.abnormis* (Fig. 3), suggesting that *E.aseptata* is a morphologically atypical (aseptate-ascospored) individual of *E.abnormis*.

Although two ITS sequences of *C.patens* (MT995055 = specimen no. FJ19131 and MW007918 = specimen no. FJ19049) were positioned in SHs dominated by *E.abnormis*, LSU and mtSSU sequences of FJ19131 and LSU sequence of FJ19049 were closely related to *Chapsa* spp. [Graphidaceae, Ostropales]. Since Lachnaceae and Graphidaceae are phylogenetically distant, the two ITS sequences MT995055 and MW007918 have been misidentified.

Considering that the monophyly of *E.abnormis* is strongly supported (Fig. 1) and members of the species share high ITS similarities (> 97%, compiled into SH1155612.08FU) (Fig. 3, Table 2), *E.abnormis* is accepted here as a species with some intraspecific morphological and phylogenetic variation.

### ﻿Taxonomy of ‘Lachnum’ palmae

*Lachnumpalmae* formed a strongly supported clade in the phylogenetic analyses (Clade II in Fig. 1). They also shared strong selectivity to palm leaves and characteristic morphology such as thick-walled asci, hairs with resinous materials and apical amorphous materials (Suppl. material 1: Fig. S2) and ectal excipulum composed of thick-walled prismatic cells and interwoven hyphae. However, sequences labeled as *L.palmae* were divided into 4 to 7 SHs in all species delimitation analyses (Fig. 3), indicating that *L.palmae* is a species complex that includes multiple potential sister species. At present, we avoid creating new species from the complex, because the morphological and ecological differences detected among SHs are not enough to delimit species boundaries, although the size of asci and ascospores differ among some SHs, as shown in Fig. 4. Phylogenetic analyses revealed that members of the *L.palmae* complex belonged to *Erioscyphella* (Fig. 1). However, we could not judge which SH within the complex is equivalent to *L.palmae* as originally described from Honduras by Kanouse (1941) and redescribed by Spooner (1987) from the type plus another specimen from New Zealand. There are no *L.palmae* sequences from the tropical American type locality, so phylogenetic characterization and recombination of the species were avoided in the present study.

## ﻿Taxonomy

### ﻿ Erioscyphella boninensis

Taxon classificationFungiHelotialesLachnaceae

Tochihara & Hosoya sp. nov.

MycoBank No: 835702

Figs 5 , 6

#### Diagnosis.

Differs from all other *Erioscyphella* species by the granulate walls of the ectal excipular cells.

**Figure 5.**
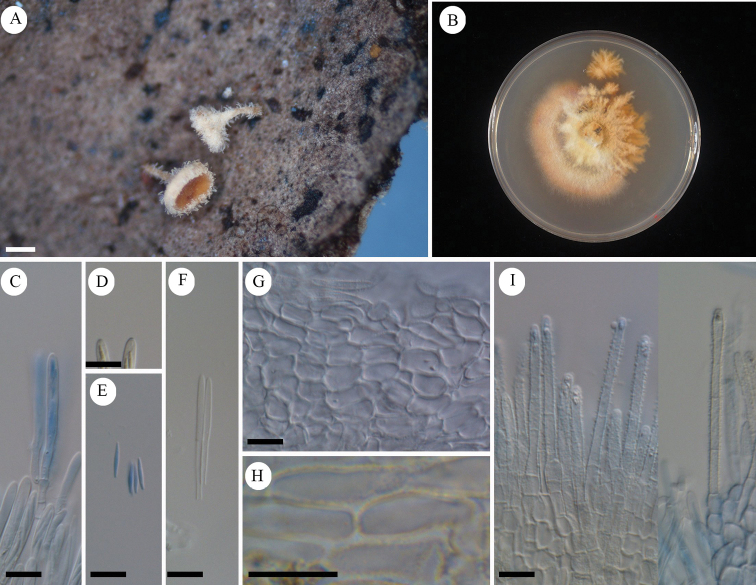
*Erioscyphellaboninensis*TNS-F-26520 (Holotype) **A** dried apothecia **B** pure culture on PDA (NBRC 114447) **C** ascus **D** ascal pore MLZ (+) **E** ascospores **F** paraphyses **G** ectal excipular cells **H** ectal excipular cells with red granules **I** hairs with resinous matters arising from ectal excipular cells. Mounted in CB/LA (**C, E–I**), MLZ (**D**). Scale bars: 1 mm (**A**); 10 µm (**C–I**).

#### Holotype.

Japan, Bonin Islands, Chichijima Island, Mt. Tsutsujiyama, 27.060556, 142.222500, ca 270 m, 28 Jun. 2009, on fallen leaves of *Pittosporumboninense*, T.Hosoya (TNS-F-26520).

**Figure 6.**
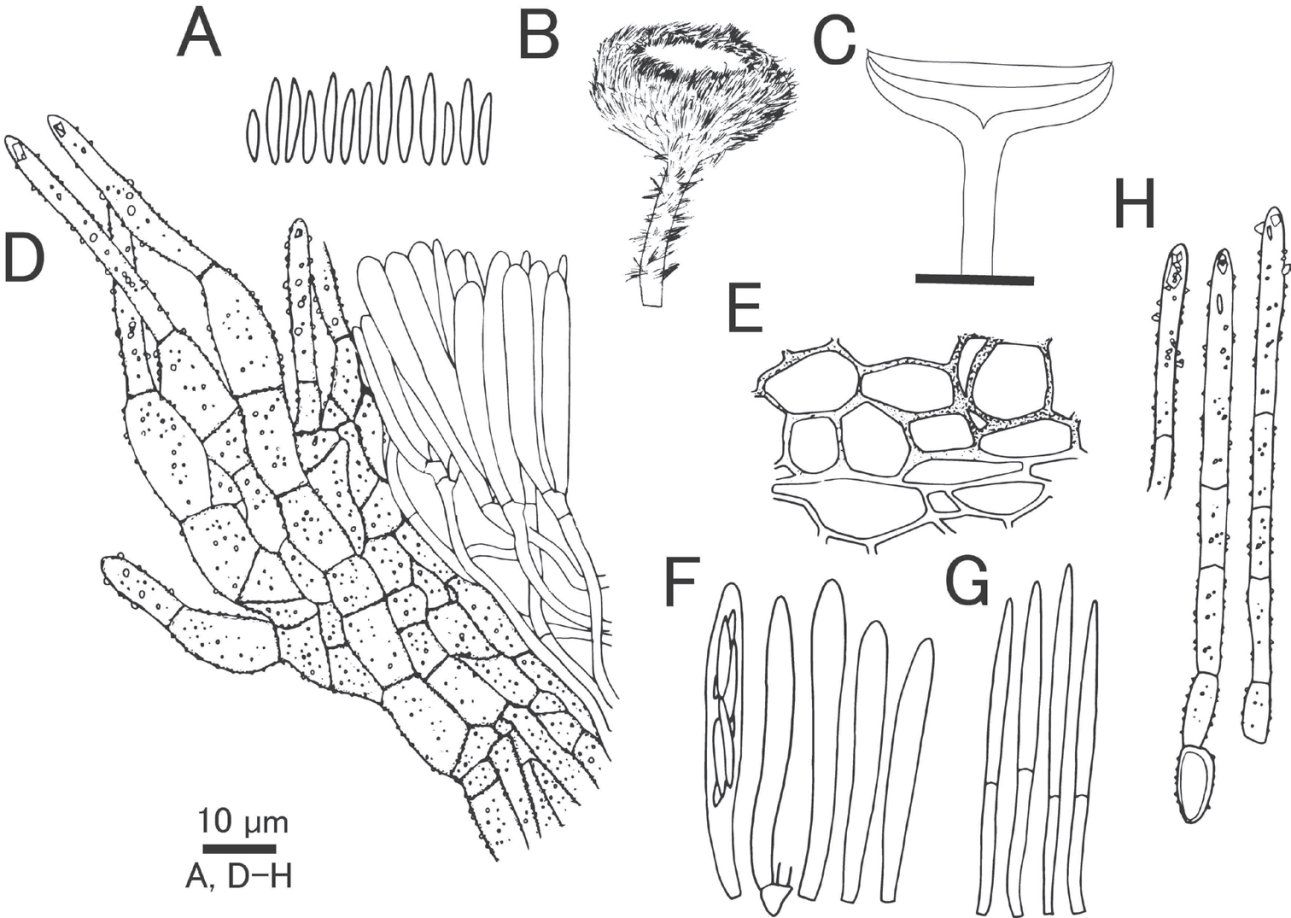
*Erioscyphellaboninensis*TNS-F-26520 (Holotype) **A** ascospores **B** apothecium **C** vertical section of an apothecium **D** expansion of a vertical section of an apothecium **E** ectal excipular cells **F** asci **G** paraphyses **H** hairs.

#### GenBank/UNITE no. ex holotype.

LC669437/UDB0779049 (ITS), LC533151 (LSU), LC533254 (mtSSU), LC533196 (RPB2).

#### Etymology.

Referring to the type locality Bonin Islands.

#### Japanese name.

Ogasawara-cha-hina-no-chawantake.

#### Description.

Apothecia scattered, superficial, 0.5–1.0 mm in diameter, having well-developed stipes, up to 1.5 mm high, cream to pale brown, externally covered with short and shiny hairs. Disc concave, cream to pale yellow. Ectal excipulum *textura prismatica* composed of long elongated cells to *textura angularis*, 6–25 × 5–13 µm, hyaline to relatively brown colored, somewhat thick-walled; cell walls covered by granules with a similar appearance to those on hairs. Stipe composed of *textura prismatica* with a granulate surface as ectal excipular cells. Medullary excipulum *textura intricata* of hyaline hyphae up to 3 µm wide. Hairs straight, cylindrical, 38–62 × 2.5–4.0 µm, hyaline, completely covered by brown granules, 2–3-septate, thin-walled, arising from swelling cells completely covered by granules; apex lacking crystals or apical amorphous materials, equipped with amber-colored resinous materials dissolvable with CB/LA at a little below the apex. Asci (36–)37.7–44(–46) × (3.5–)3.6–4.2(–4.5) µm (av. 41 ±3.2 × 3.9 ± 0.3 µm, n = 16), 8-spored, cylindrical-clavate; pore blue in MLZ without 3% KOH pretreatment; croziers absent at the basal septa. Ascospores (9–)10–12.3(–13) × 1.2–1.7(–1.8) µm (av. 11 ± 1.2 × 1.5 ± 0.2 µm, n = 16), Q = (6.3–)6.9–9.2(–10) (av. 7.8 ± 1.5, n = 16), fusiform, aseptate. Paraphyses straight, up to 2.5 µm wide, septate, exceeding the asci up to 5 µm, narrowly lanceolate.

#### Culture characteristics.

Colony of NBRC 114447/TNS-F-26520 on PDA umbonate forming a dome-shape, slightly sulcate. Context not shiny, velvety, buff at the center, paler toward the margin, dark buff from the reverse. Sectors and zonation absent. Aerial mycelium white or buff, dense cottony, forming white mycelium strands except in the margin. Margin distinct, entire, flat. Asexual morph absent.

#### Distribution.

Japan. (Bonin Islands). Known only from the type locality.

#### Notes.

Granulation on the surface of the ectal excipular cells has been observed only in *Incrucipulum* in Lachnaceae (Baral and Krieglsteiner 1985; Tochihara and Hosoya 2019), and *E.boninensis* is the first report for such a character in *Erioscyphella* (Fig. 5H, 6E). Phylogenetic analysis revealed that *E.boninensis* is closely related to *E.paralushanensis* (Fig. 1). The two species (Clade IA, Fig. 1) have colored granules on hairs and forming red mycelia on PDA. However, granulation of ectal excipulum is seen only in *E.boninensis*.

### ﻿ Erioscyphella hainanensis

Taxon classificationFungiHelotialesLachnaceae

(W.Y. Zhuang and Zheng Wang) Hosoya and Tochihara comb. nov.

MycoBank No: 835707

≡ Lachnumhainanense W.Y. Zhuang & Zheng Wang, Mycotaxon 67: 25 (1998).

#### Diagnosis.

Forming apothecia with long stipes and long hairs. Differing *E.sinensis* in much shorter ascospores.

#### Japanese name.

Shii-Kashi-hina-no-chawantake.

#### Specimens examined.

Japan, Niigata, Minamiuonuma, 37.056808, 138.80705, ca 720 m, 14 May 2010, on fallen leaves of *Quercusglauca*, T.Hosoya (TNS-F-35049). Ibid (TNS-F-35056). Japan, Kanagawa, Hiratsuka, 35.33861111, 139.285, ca 80 m, 12 Apr. 2015, on fallen leaves of *Q.myrsinifolia*, M.Nakajima (TNS-F-61775). JAPAN, Kanagawa, Kamakura, 35.30756, 139.51958, ca 40 m, 24 Apr. 2015, on fallen leaves of *Q.serrata*, M.Nakajima (TNS-F-61941). Japan, Gunma, Midori, 36.476684, 139.242771, ca 510 m, 9 May 2016, on fallen leaves of *Q.serrata*, K.Furuya (TNS-F-65722). Japan, Kanagawa, Hiratsuka, 35.340139, 139.287167, ca 60 m, 18 May 2017, on fallen leaves of *Q.glauca*, Y.Tochiara (TNS-F-80356). The same locality, on fallen leaves of *Castanopsissieboldii*, Y. Tochihara (TNS-F-80371).

#### Distribution.

China (Hainan), Japan (Honshu: Kanto region).

#### Notes.

Based on the UNITE SH system at a 3% threshold, ITS sequences of this species were integrated into a single SH (DOI: SH1155844.08FU). SH1155844.08FU included sequences labeled as ‘Hyaloscyphaceae’ (JX984680) in UNITE and ‘*L.albidulum*’ (MK282242) in INSDC (Table 2). JX984680 was sequenced from air samples in Seoul, South Korea, and was not tied to any fungal specimens or cultures. *Lachnumalbidulum* is common on leathery dicot leaves of the old and new world tropics (Haines 1992). *Erioscyphellahainanensis* resembles *L.albidulum* in morphology, but *L.albidulum* has yellow resinous substances at the tip of apothecial hairs and occurs on dead leaves of Rubiaceae (Haines 1992), whereas *E.hainanensis* lacks resinous substances and occurs on leaves of broad-leaved trees (Zhuang and Wang 1998b; Hosoya et al. 2013). Therefore, we presume that MK282242, coexisting with *L.hainanense* in every SH, was misidentified as *L.albidulum*. No sequences are available for *L.albidulum* specimens from the type locality. *Lachnumhainanense* was therefore judged as acceptable species, and recombined into *Erioscyphella*.

*Erioscyphellahainanensis* resembles *E.sinensis* in occurring on dead leaves of *Quercus* spp. or *Castanopsis* spp. However, *E.hainanensis* has much shorter ascospores than *E.sinensis*. In this study, presence of minute, hyaline apical amorphous materials and absence of any crystals or resinous materials were confirmed in both species (Suppl. material 1: Fig. S2).

### ﻿ Erioscyphella insulae

Taxon classificationFungiHelotialesLachnaceae

Tochihara & Hosoya sp. nov.

MycoBank No: 835703

Figs 7 , 8

#### Diagnosis.

Characterized by pure white apothecia unlike related species *Lachnumnothofagi*, and two-layered ectal excipulum.

#### Holotype.

Japan, Okinawa, Yaeyama, Taketomi, Iriomote Island, Otomi, 24.297458, 123.866128, ca 50 m, 12 Jun. 2011, on fallen bark of unidentified tree, T.Fukiharu (TNS-F-39720).

#### GenBank/UNITE no. ex holotype.

LC669451/UDB0779063 (ITS), LC533177 (LSU), LC533261 (mtSSU), LC533207 (RPB2).

#### Other specimens examined.

Japan, Bonin Islands, Hahajima Island, Sekimon, 26.666686, 142.152222, ca 260 m, 24 Jun. 2009, on fallen bark of unidentified tree, T.Hosoya (TNS-F-26485, 26500).

#### Etymology.

Referring to the occurrence of the species on remote islands in Japan.

#### Japanese name.

Shima-hina-no-chawantake.

#### Description.

Apothecia gregarious, superficial, 0.7–1.4(–2.5) mm in diameter, short- and thick-stipitate, up to 0.8 mm high, externally white to cream throughout but sometimes pale brown in the lower parts, covered with white hairs. Disc concave, cream to pale yellow (fresh state not observed). Ectal excipulum composed of two layers: outer layer *textura angularis*, up to 20 µm thick, 3–28 × 2–8 µm, hyaline, thin to relatively thick-walled, with cell walls smooth; inner layer up to 15 µm thick, *textura porrecta* composed of hyaline hyphae up to 5 µm wide. Medullary excipulum up to 100 µm thick, composed of hyaline hyphae forming *textura intricata*; hyphae up to 3 µm wide. Hairs straight or irregularly curved, cylindrical, sometimes branched, up to 125 × 2.5–3.0 µm, hyaline, completely granulate, thin-walled; lacking crystals or resinous materials; apex usually equipped with hyaline apical amorphous materials. Asci (88–)92–101(–106) × 6–7.3(–8) µm (av. 96 ± 4.5 × 6.7 ± 0.6 µm, n = 18), 8-spored, thick-walled, cylindrical-clavate, arising from ascogenous hyphae branching several times; pore blue in MLZ without 3% KOH pretreatment; croziers absent at the basal septa. Ascospores (24–)26.7–34.5(–39) × (1.8–)1.9–2.3(–2.5) µm (av. 31 ± 3.9 × 2.1 ± 0.2 µm, n = 18), Q = (11–)12.5–17(–20) (av. 14.7 ± 2.3, n = 18), showing various shapes and lengths, usually long fusiform and sometimes hypsiloid or sigmoid due to bending of both ends, sometimes swelling or constricted irregularly, aseptate or one- to three-septate (usually one-septate). Paraphyses straight, narrowly lanceolate, up to 2.5 µm wide, septate, exceeding the asci up to 7.5 µm.

#### Culture characteristics.

Colony of NBRC 114445/TNS-F-26500 and NBRC 114459/TNS-F-39720 on PDA relatively thick-planar, pruinose, white to cream, ivory at the margin, pale sepia. Sectors and zonation absent. Aerial mycelium white to pale ocher, mainly developed except in the margin, not forming mycelial strands. Soluble pigment amber colored produced at the center. Margin unclear, flat and immersed into agar, radially undulate. Anamorph not seen.

#### Distribution.

Japan (Bonin Islands, Yaeyama Islands).

#### Notes.

**Figure 7.**
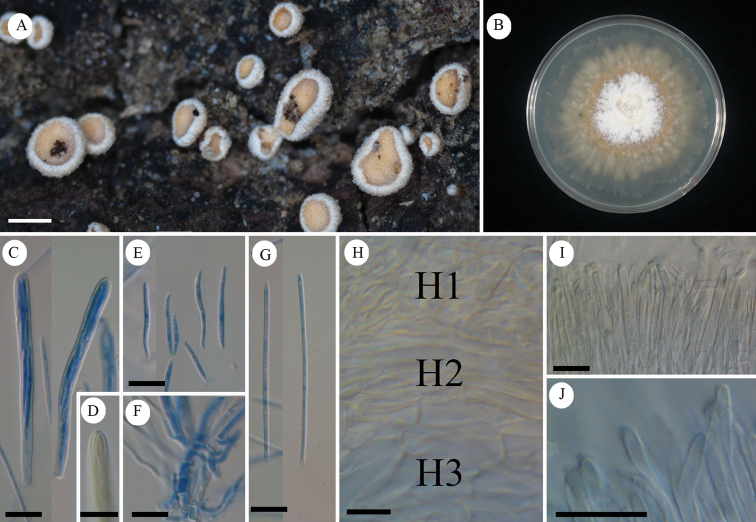
*Erioscyphellainsulae*TNS-F-39720 (Holotype) **A** dried apothecia **B** a pure culture on PDA (NBRC 114459) **C** asci **D** ascal pore MLZ (+) **E** ascospores **F** ascogenous hyphae **G** paraphyses **H** layer structures of excipulum **H1** medullary excipulum **H2** inner layer of ectal excipulum composed of hyphae **H3** outer layer of ectal excipulum composed of *textura angularis***I, J** hairs with apical amorphous materials. Mounted in CB/LA (**C, E–J**), MLZ (**D**). Scale bars: 1 mm (**A**); 10 µm (**A–J**).

**Figure 8.**
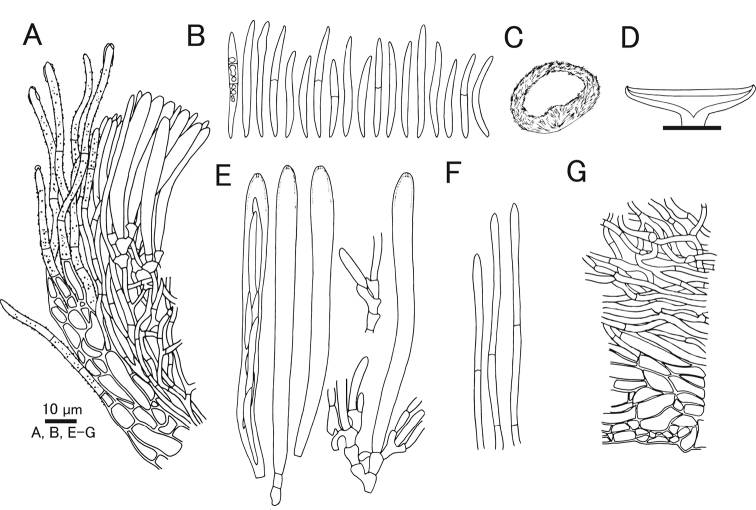
*Erioscyphellainsulae*TNS-F-39720 (Holotype) **A** expansion of a vertical section of an apothecium **B** ascospores **C** apothecium **D** vertical section of an apothecium **E** asci **F** paraphyses **G** layer structures of excipulum.

This fungus resembles *Lachnumnothofagi* (Dennis) Spooner in the size and shape of apothecia, ascospores, asci, and hairs. However, *E.insulae* has completely hyaline hairs and ectal excipulum, and hairs are equipped with apical materials (Fig. 7J, 8A), whereas *L.nothofagi* has partly to totally brown hairs and ectal excipulum (Spooner 1987). *Lachnumnothofagi* is currently known only from New Zealand and Australia and mainly arises from *Nothofagus* spp., which are native in the southern hemisphere (Spooner 1987).

### ﻿ Erioscyphella otanii

Taxon classificationFungiHelotialesLachnaceae

Tochihara sp. nov.

MycoBank No: 835704

Figs 9 , 10

#### Diagnosis.

Characterized by pure white minute apothecia (< 0.3 mm in diameter) unlike *L.diminutum* with rather colored apothecia, and smaller asci compared to similar species *Lachnumminutum*.

**Holotype.** Japan, Hokkaido, Horonobe, Toikambetsu, Teshio Experimental Forest, Field Science Center for Northern Biosphere, Hokkaido University, 44.993978, 142.130125, ca 400 m, 11 Jul. 2018, on fallen leaves of *Sasasenanensis*, Y.Tochihara & K.Kaneko (TNS-F-81472).

#### GenBank/UNITE no. ex holotype.

LC669471/UDB0779083 (ITS), LC533179 (LSU), LC533286 (mtSSU), LC533226 (RPB2).

#### Other specimen examined.

Japan, Hokkaido, Sapporo, Mt. Moiwa, 43.024718, 141.318427, ca 530 m, 21 Jun. 1965, on fallen leaves of *Sasakurilensis*, Y.Otani (TNS-F-50482, in poor condition).

#### Etymology.

Referring to the name of Dr Yoshio Otani, the first discoverer of this species.

#### Japanese name.

Kita-sasaba-hina-no-chawantake.

#### Description.

Apothecia scattered, superficial, minute, 0.1–0.3 mm in diameter, at first spherical and later urceolate, having well-developed stipes, up to 0.3 mm high, pure white, externally covered with short white hairs, never colored brown. Disc concave, almost enclosed by an incurving margin when fresh and dry, cream to pale yellow when dry (not observed when fresh). Ectal excipulum *textura prismatica* like stone pavings arranged in rows, 3–25 × 3–8 µm, hyaline, relatively thick-walled; cell walls smooth. Medullary excipulum *textura intricata*; hyphae up to 2.5 µm wide. Hairs straight, cylindrical or tapering toward the apices, up to 60 µm long, up to 5 µm wide near the bases and 2.5–3.0 µm wide near the apices, arising from swollen ectal excipular cells, hyaline, up to 3-septate (usually 1- or 2-septate), thin-walled, completely granulated; granules dense near the apices and coarse toward the bases; apex sometimes with a hyaline and inconspicuous apical amorphous materials not dissolved with CB/LA, lacking any crystals or resinous materials. Asci (33–)34–38.8(–41) × 4–5 µm (av. 37 ± 2.2 × 4.4 ± 0.4 µm, n = 15), 8-spored, cylindrical-clavate, relatively thick-walled; pore blue in MLZ without 3% KOH pretreatment; croziers absent at the basal septa. Ascospores (11.5–)12.3–14.6(–15) × (1.2–)1.36–1.7(–1.8) µm (av. 13.4 ± 1.2 × 1.6 ± 0.2 µm, n = 15), Q = (6.7–)7.8–9.6(–10.8) (av. 8.7 ± 0.9, n = 15), fusiform, aseptate. Paraphyses straight, narrowly lanceolate to lanceolate, up to 2.5 µm wide, septate, exceeding the asci up to 10 µm.

#### Culture characteristics.

Colony of NBRC 114476/TNS-F-81472 on PDA flat, partially protruding and forming mycelial mass, divided into two sectors. One sector flat, wooly to velvety, white to cream; dark ocher from the reverse. The other sector with wooly context, white and partly yellow; pale ocher from the reverse. Aerial mycelia developed throughout the colony, white, sparse to cottony, not forming mycelium strands. Margin distinct, flat and immersed into the agar. Soluble pigment absent. Asexual morph absent.

#### Distribution.

Japan (Hokkaido; subarctic zone).

#### Notes.

**Figure 9.**
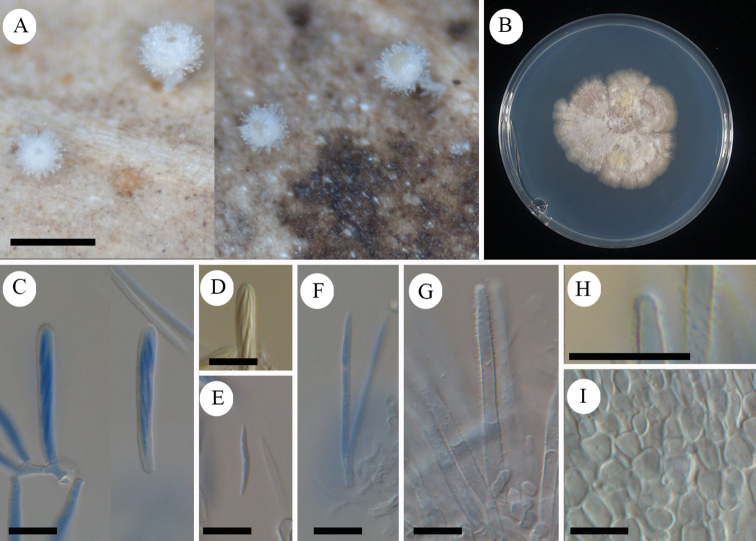
*Erioscyphellaotanii*TNS-F-81472 (Holotype) **A** dried apothecia **B** pure culture on PDA (NBRC 114476) **C** asci **D** ascal pore MLZ (+) **E** ascospore **F** paraphyses **G** a hair **H** hair-apex with a apical amorphous material **I** ectal excipular cells. Mounted in CB/LA (**C, E–I**), MLZ (**D**). Scale bars: 0.5 mm (**A**); 10 µm (**C–I**).

**Figure 10.**
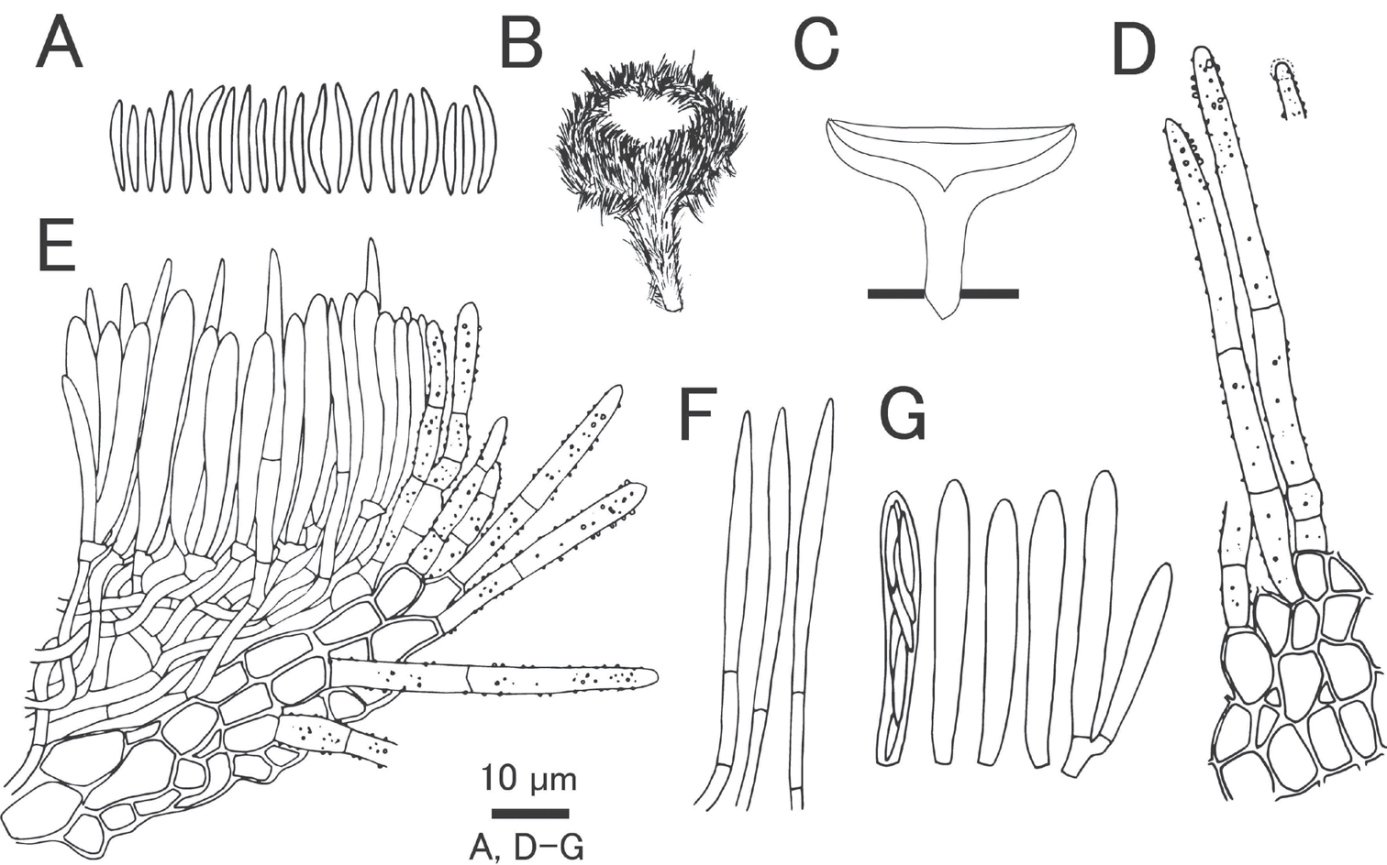
*Erioscyphellaotanii*TNS-F-81472 (Holotype) **A** ascospores **B** apothecium **C** vertical section of an apothecium **D** hairs with cap-like structures arising from ectal excipular cells **E** expansion of a vertical section of an apothecium **F** paraphyses **G** asci.

*Erioscyphellaotanii* was first collected and documented by Otani (1967) under the misapplied name *Dasyscyphusdiminutus* (TNS-F-50482). Based on the description, we concluded that the specimen was the same species as TNS-F-81472. The present species is very similar to *Lachnumdiminutum* (Roberge ex Desm.) Rehm in the minute apothecia, ascospore size, and narrow paraphyses; however, *E.otanii* is pure white when fresh and dry (Fig. 9A, in dried state) and occurs on bamboo leaves, while *L.diminutum* is somewhat brown in the exterior parts of apothecia and occurs on sheaths of *Juncus* spp. (Dennis 1949). In the mature state, the apothecia of *E.otanii* become urceolate (Fig. 9A and Fig. 10B), whereas the apothecia of *L.diminutum* are flat (Dennis 1949). The ITS sequence of TNS-F-81472 showed low similarity (< 80%) with that of *L.diminutum* collected in France (GenBank accession number: MH857306). Based on the French sequence, *L.diminutum* is phylogenetically a good *Lachnum*.

The appearance of *E.otanii* is also similar to that of the graminicolous species *Lachnumminutum* W.Y. Zhuang and M. Ye documented in China (Ye and Zhuang 2003). *Erioscyphellaotanii* is distinguished from *L.minutum* in having smaller asci, although DNA sequences of the species are not available.

### ﻿ Erioscyphella papillaris

Taxon classificationFungiHelotialesLachnaceae

Tochihara sp. nov.

MycoBank No: 835705

Figs 11 , 12

#### Diagnosis.

Characterized by protruding papillary hairs with hyaline apical amorphous materials.

#### Holotype.

Japan, Gunma, Minakami, Yubiso, Mt. Tanigawadake, 36.064014, 141.344653, ca 710 m, 16 Jul. 2017, on both sides of a fallen leaf of bamboo, Y.Tochihara (TNS-F-81272).

#### GenBank/UNITE no. ex holotype.

LC669473/UDB0779085 (ITS), LC533161 (LSU), LC533285 (mtSSU), LC533204 (RPB2).

#### Etymology.

Referring to papillate hair apices.

#### Japanese name.

Sasaba-hina-no-chawantake.

#### Description.

Apothecia gregarious, superficial, minute, 0.1–0.3 mm in diameter, short-stipitate, up to 0.25 mm high, externally densely covered with pure white short hairs. Disc concave, white to lemon yellow when fresh and dry. Ectal excipulum *textura prismatica* composed of cuboid cells, 3–13 × 2.5–7 µm, hyaline, thin-walled, lacking carotenoid pigments; cell walls smooth. Medullary excipulum *textura intricata* of hyaline hyphae up to 3 µm wide. Hairs straight, cylindrical, 45–75 × 3–5 µm, 2–3-septate, hyaline, totally granulate, thin-walled, arising from swollen cells; apical cells rather longer than other cells, 30–40 µm long, with papillate at the apex, sometimes swelling, equipped with hyaline and globose apical amorphous materials not dissolved with CB/LA, lacking any crystals or resinous matters. Asci (59–)59.8–66(–69) × (7.5–)7.6–8.3(–9) µm (av. 63 ± 2.9 × 8.0 ± 0.4 µm, n = 16), 8-spored, cylindrical-clavate; pore inamyloid with MLZ without 3% KOH pretreatment, faint blue with MLZ with 3% KOH pretreatment, dark blue with IKI with and without KOH pretreatment; vesicle apparatus inverted-v-shaped present near the apices; croziers absent at the basal septa; base sympodially branched. Ascospores (16–)17.5–21.7(–24) × (2–)2.3–2.8(–3) µm (av. 20 ± 2.1 × 2.6 ± 0.3 µm, n = 20), Q = (6.4–)6.8–8.9(–9.8) (av. 7.8 ± 1.0, n = 20), fusiform, aseptate, or one-septate (rarely two-septate), filled with hyaline oil drops. Paraphyses straight, cylindrical, up to 3 µm wide, septate, containing small hyaline lipid bodies, equal or scarcely exceeding the asci.

#### Culture characteristics.

Colony of NBRC 113937/TNS-F-81272 on PDA divided into two semicircular zones. The first zone umbonate, pruinose, white, producing white aerial mycelia densely, presenting wooly appearance; margin distinct, entire, flat. The second zone flat, glutinous, white to beige with concentric patterns, producing few aerial mycelia; margin entire, flat and immersed into agar, irregularly undulate. The reverse uniform unrelated to the zoning position, beige to pale dark brown throughout. Soluble pigment and asexual morph absent throughout the colony.

#### Distribution.

Japan (Mt. Tanigawa). Currently known only from the type locality.

#### Notes.

**Figure 11.**
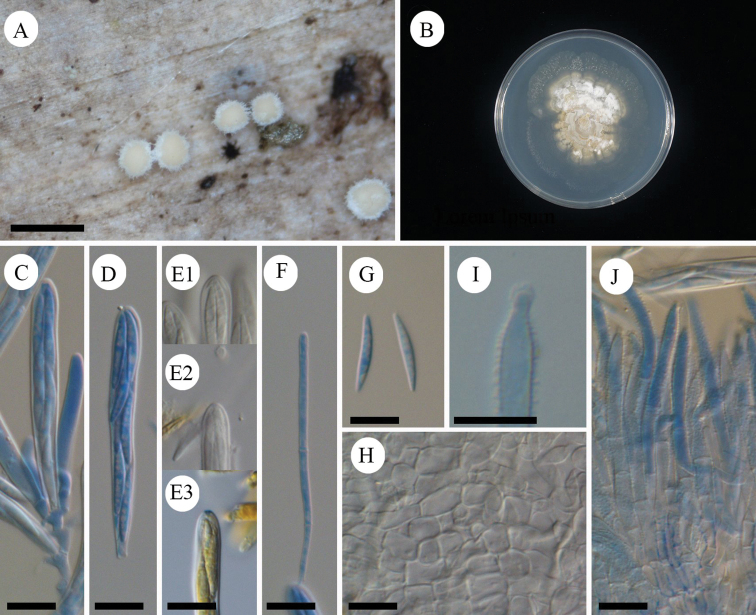
*Erioscyphellapapillaris*TNS-F-81272 (Holotype) **A** dried apothecia **B** pure culture on PDA (NBRC 113937) **C** Ascus arising from ascogenous hyphae **D** an ascus **E** ascal pore iodine reactions **E1**MLZ (-) without 3% KOH pretreatment **E2**MLZ (-) with 3% KOH pretreatment **E3** IKI (+) without 3% KOH pretreatment **F** paraphysis **G** ascospores with guttules **H** ectal excipulum **I** hair-apex with a apical amorphous material **J** hairs. Mounted in CB/LA (**C, D, F–J**), MLZ (**E1, E2**), IKI (**E3**). Scale bars: 0.5 mm (**A**); 10 µm (**C–J**).

**Figure 12.**
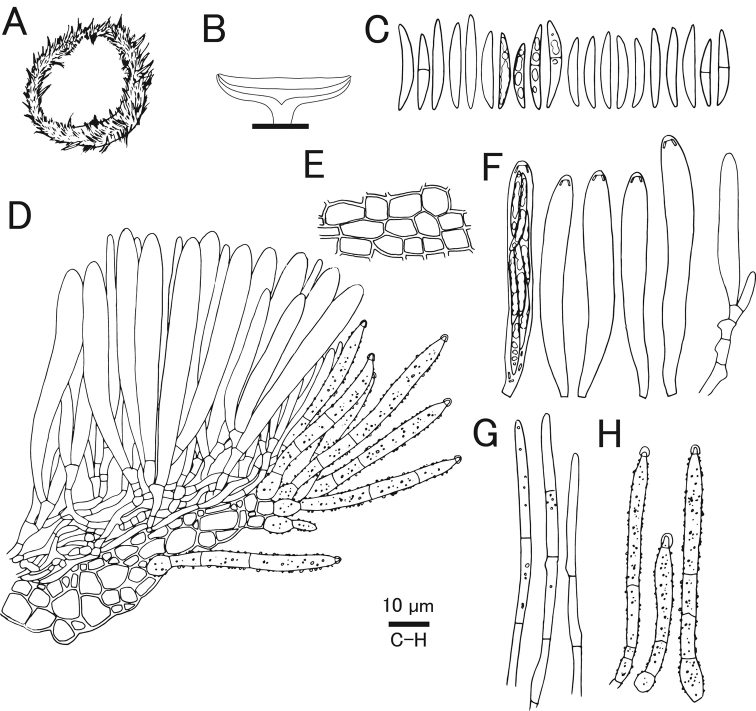
*Erioscyphellapapillaris*TNS-F-81272 (Holotype) **A** apothecium **B** vertical section of an apothecium **C** ascospores **D** expansion of an vertical section of an apothecium **E** ectal excipular cells **F** asci **G** paraphyses **H** hairs with cap-like structures.

This species is similar to Lachnumsclerotiivar.microascum in the dimension and shape of asci and ascospores, habitats, and inconspicuous ascus apex reaction in MLZ (Zhuang 2004). However, *E.papillaris* has ascospores containing conspicuous guttules in any mount (Fig. 11G) and filiform paraphyses rarely exceeding the asci (Fig. 11F, Fig. 12D, and Fig. 12G), whereas L.sclerotiivar.microascum has non-guttulate asci and narrowly lanceolate to lanceolate paraphyses exceeding the asci by 15–18 µm (Zhuang 2004). Although DNA sequences of L.sclerotiivar.microascum are not available, we judged the present fungus as different from it, because the presence or absence of guttules in ascospores is a significant taxonomic character at the species level (Baral 2015).

Papillate hairs are also shown in the line drawings of *Lachnumgahniae* Spooner (Spooner 1987), suggesting the relationship of the present fungus to Australasian species. However, *L.gahniae* can be distinguished by having longer hairs, occurring on different substrates (leaves of Cyperaceae) and showing different ascal-iodine reactions (MLZ+) (Spooner 1987), although DNA sequences of *L.gahnia* are not available.

### ﻿ Erioscyphella paralushanensis

Taxon classificationFungiHelotialesLachnaceae

Tochihara and Hosoya sp. nov.

MycoBank No: 839618

Figs 13 , 14

#### Diagnosis.

Characterized by throughout red apothecia occurring on bamboo sheaths. Similar to *E.lushanensis* in macro- and micromorphology and habitats, but has larger asci and ascospores.

#### Holotype.

Japan, Shizuoka, Atami, Izusan, 35.128834, 139.051194, ca 620 m, 8 Jun. 2015, on fallen sheaths of *Pleioblastusargenteostriatus*, M.Nakajima (TNS-F-61920).

#### GenBank/UNITE no. ex holotype.

LC669463/UDB0779075 (ITS), LC533141 (LSU), LC533267 (mtSSU), LC533220 (RPB2).

#### Etymology.

Referring to the similarity with *E.lushanensis*.

#### Japanese name.

Akage-hina-no-chawantake.

#### Description.

Apothecia scattered, superficial, 0.7–1.5 mm in diameter, long-stipitate, up to 2.0 mm high, externally covered with dark-red hairs. Disc concave, cream to pale yellow. Ectal excipulum well-developed *textura prismatica* and partly *t. angularis*, 6–13 × 2.0–2.5 *µm*, *hyaline*, *relatively* thick-walled, with smooth walls. Medullary excipulum *textura intricata* of hyaline hyphae up to 2 µm wide. Hairs straight, cylindrical, up to 160 *µm long*, *2.0*–3.0 µm wide, pale brown but hyaline near the bases; hair cells narrowly septate, > 7 µm long, covered by big and amber-colored granules; granules big and dense near the apices and smaller and sparse near the bases, up to 2 µm in diameter near the apices, equipped with amber-colored resinous materials that dissolves in CB/LA at any position of hairs; apices with amber-colored apical amorphous materials, lacking any crystals. Asci (59–)61.4–70.2(–73) × (4.5–)4.7–5.6(–6) µm (av. 65.8 ± 4.4 × 5.2 ± 0.4 µm, n = 15), Q = (11.5–)12–13.6(–14.6) (av. 12.8 ± 0.8, n = 15), 8-spored, cylindrical-clavate; pore faintly blue in MLZ without 3% pretreatment, clear blue in MLZ with 3% KOH pretreatment and IKI without 3% KOH pretreatment. Ascospores (14–)15.8–20.7(–22) × (1.5–)1.7–2.0 µm (av. 18.2 ± 2.5 × 1.8 ± 0.2 µm, n = 15), Q = (7.5–)8.7–11.2(–12.6) (av. 9.9 ± 1.3, n = 15), septate, sometimes bent to U-shaped or S-shaped, containing conspicuous guttules; guttules hyaline but sometimes red. Paraphyses straight, up to 2 µm wide, septate, exceeding the asci 5–10 *µm*, initially cylindrical to clavate, later becoming narrowly lanceolate.

#### Culture characteristics.

Colony of NBRC 114468/TNS-F-61920 on PDA flat, sparse, dendritically spread. Context wooly, ocher to pale buff, dark buff from the reverse. Sectors and zonation absent. Aerial mycelium ocher to pale buff, dense cottony, developed near the center, forming white mycelium strands; margin distinct, flat and partly immersed into the agar. Asexual morph absent. Soluble pigments present, buff, dyeing agar without colony pale buff.

#### Distribution.

Japan (Shizuoka). Currently known only from the type locality.

**Figure 13.**
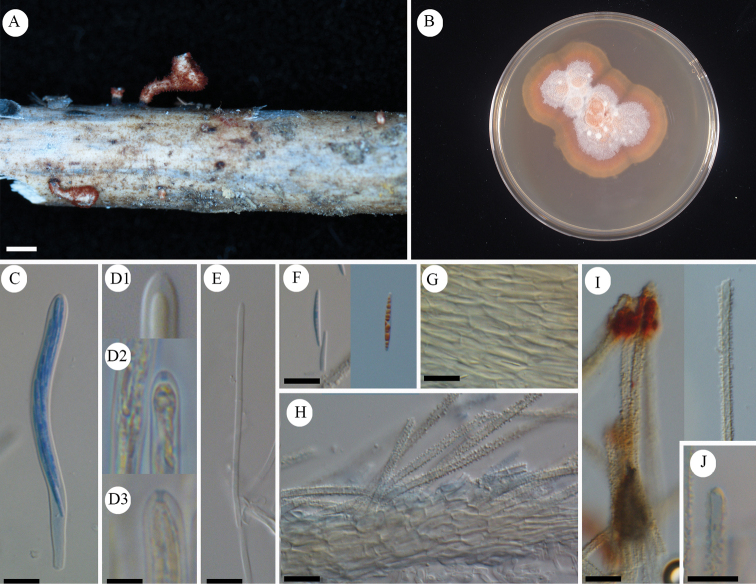
*Erioscyphellaparalushanensis*TNS-F-61920 (Holotype) **A** apothecia **B** pure culture on PDA (NBRC 114468) **C** ascus **D** ascal pore iodine reactions **D1**MLZ (faintly +) without 3% KOH pretreatment **D2**MLZ (+) with 3% KOH pretreatment **D3** IKI (+) without 3% KOH pretreatment **E** paraphysis **F** ascospores **G** ectal excipular cells **H** marginal section of an apothecium generating hairs **I** hairs with red resinous materials **J** apical amorphous materials of hairs. Mounted in CB/LA (**C, E–J**), MLZ (**D1, D2**), IKI (**D3**). Scale bars: 0.5 mm (**A**); 10 µm (**C–J**).

#### Notes.

**Figure 14.**
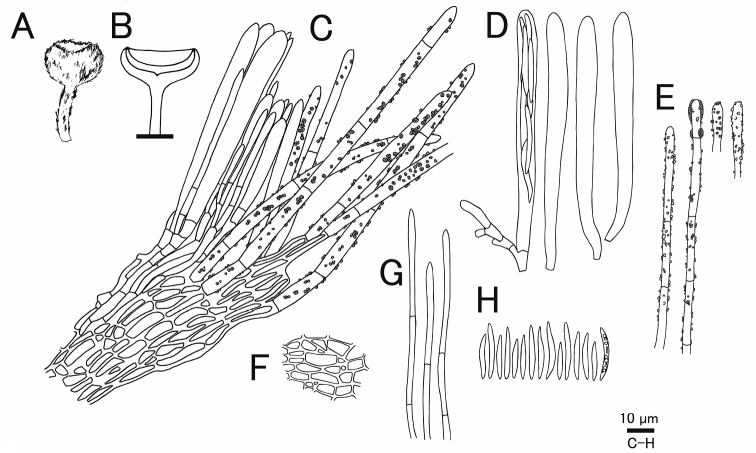
*Erioscyphellaparalushanensis*TNS-F-61920 (Holotype) **A** apothecia **B** vertical section of an apothecium **C** expansion of an vertical section of an apothecium **D** asci **E** hairs **F** ectal excipulum **G** paraphyses **H** ascospores.

*Erioscyphellaparalushanensis* is closely related to *E.lushanensis* in having red hairs (Fig. 13I) and the ectal excipulum composed of well-developed rectangular cells in common (Fig. 13H, Fig. 14C, and Fig. 14F) (Zhuang and Wang 1998a). Compared with *E.lushanensis*, *E.paralushanensis* has slightly larger asci, ascospores and hairs. Red guttules in ascospores were observed only in *E.paralushanensis* (Fig. 13F). In this study, we proposed the present fungus as a new species, because species delimitation analyses based on ITS sequences strongly supported that *E.paralushanensis* is different from *E.lushanensis* (Fig. 3).

### ﻿ Erioscyphella sasibrevispora

Taxon classificationFungiHelotialesLachnaceae

Tochihara & Hosoya sp. nov.

MycoBank No: 835706

Figs 15 , 16

#### Diagnosis.

Characterized by wooly appearance and yellow to orange discs, and distinguished from similar species Lachnumnovoguineensevar.yunnanicum in having shorter ascospores.

#### Holotype.

Japan, Hokkaido, Tomakomai, Utonai, 42.705314, 141.7346, ca 10 m, 16 Jun. 2018, on fallen sheaths of *Sasanipponica*, Y.Tochihara & T.Hosoya (TNS-F-81401).

#### GenBank/UNITE no. ex holotype.

LC669470/UDB0779082 (ITS), LC533174 (LSU), LC533269 (mtSSU), LC533217 (RPB2).

#### Other specimen examined.

Japan, Gunma, Higashiagatsuma, 36.562253, 138.724139, ca 1330 m, 6 Jun. 2017, on fallen sheaths of *Sasaveitchii*, Y.Tochihara & T.Hosoya (TNS-F-80399, in bad condition).

#### Etymology.

“sasi” means bamboo [host plants] and “brevispora” means shorter ascospores compared to L.novoguineensevar.yunnanicum.

#### Japanese name.

Sasa-no-youmou-chawantake.

#### Description.

Apothecia gregarious, superficial, 0.6–1.3 mm in diameter, short-stipitate, up to 0.8 mm high, pure white, externally covered with long white hairs. Disc concave, yellow to pale orange when fresh and dry. Ectal excipulum *textura prismatica* to *t. angularis*, 3–16 × 2–10 µm, hyaline, thin-walled; surface smooth. Medullary excipulum *textura intricata* of hyaline hyphae up to 2 µm wide. Hairs straight, delicate, cylindrical with relatively acute apices, up to 190 × 2–3 µm, hyaline, totally granulate, thin-walled; apical cell a little longer than other cells, lacking any crystals, resinous materials, or apical amorphous materials. Asci (79–)82.5–90(–95) × (6–)6.6–8.1(–9) µm (av. 86 ± 4.0 × 7.4 ± 0.8 µm, n = 15), 8-spored, cylindrical-clavate; lateral parts sometimes swelling irregularly; pore blue in MLZ without 3% KOH pretreatment; croziers with perforation present at the basal septa. Ascospores (26–)27.9–36.1(–39) × (1.5–)1.7–2 µm (av. 32 ± 4.1 × 1.8 ± 0.2 µm, n = 17), Q = (13–)15–19.7(–21) (av. 17.5 ± 2.3, n = 17), long fusiform, usually 3-septate, rarely 0- to 2-septate (only observed in TNS-F-81401 because TNS-F-80399 was immature). Paraphyses straight, lanceolate, 2.5–4 µm wide, densely septate, exceeding the asci up to 15 µm. Note that the description is solely based on the holotype because another examined specimen TNS-F-80399 was in bad condition.

#### Culture characteristics.

Colony of NBRC 114475/TNS-F-81401 on PDA wrinkled. Context cottony and partially funiculose, white, turning ocher at the center; almost ocher except for the white margin from the reverse. Sectors and zonation absent. Aerial mycelium developed throughout the colony, concolous, forming mycelium strands. Margin indistinct, flat and immersed into agar. Soluble pigment absent. Asexual morph absent.

#### Distribution.

Japan (cool-temperate zone, subarctic zone).

#### Notes.

**Figure 15.**
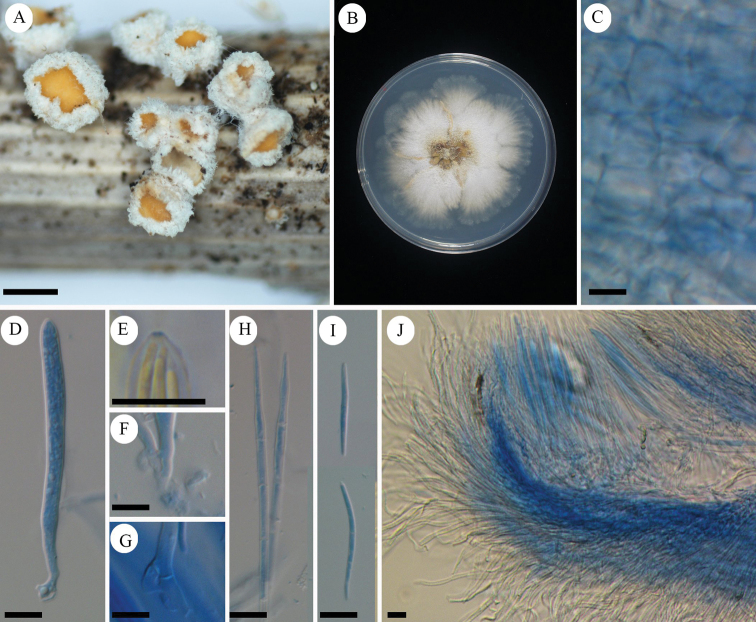
*Erioscyphellasasibrevispora*TNS-F-81401 (Holotype, **A–F, H–J**). Lachnumnovoguineensevar.yunnanicumTNS-F-16442 (**G**) **A** dried apothecia **B** a pure culture on PDA (NBRC 114475) **C** ectal excipular cells **D** ascus **E** an ascal pore MLZ (+) **F** ascal base with a perforated crozier **G** ascal base with a perforated crozier **H** septated paraphyses **I** ascospores **J** vertical section through the apothecium. Mounted in CB/LA (**D, F–J**), MLZ (**E**). Scale bars: 1 mm (**A**); 10 µm (**C–J**).

**Figure 16.**
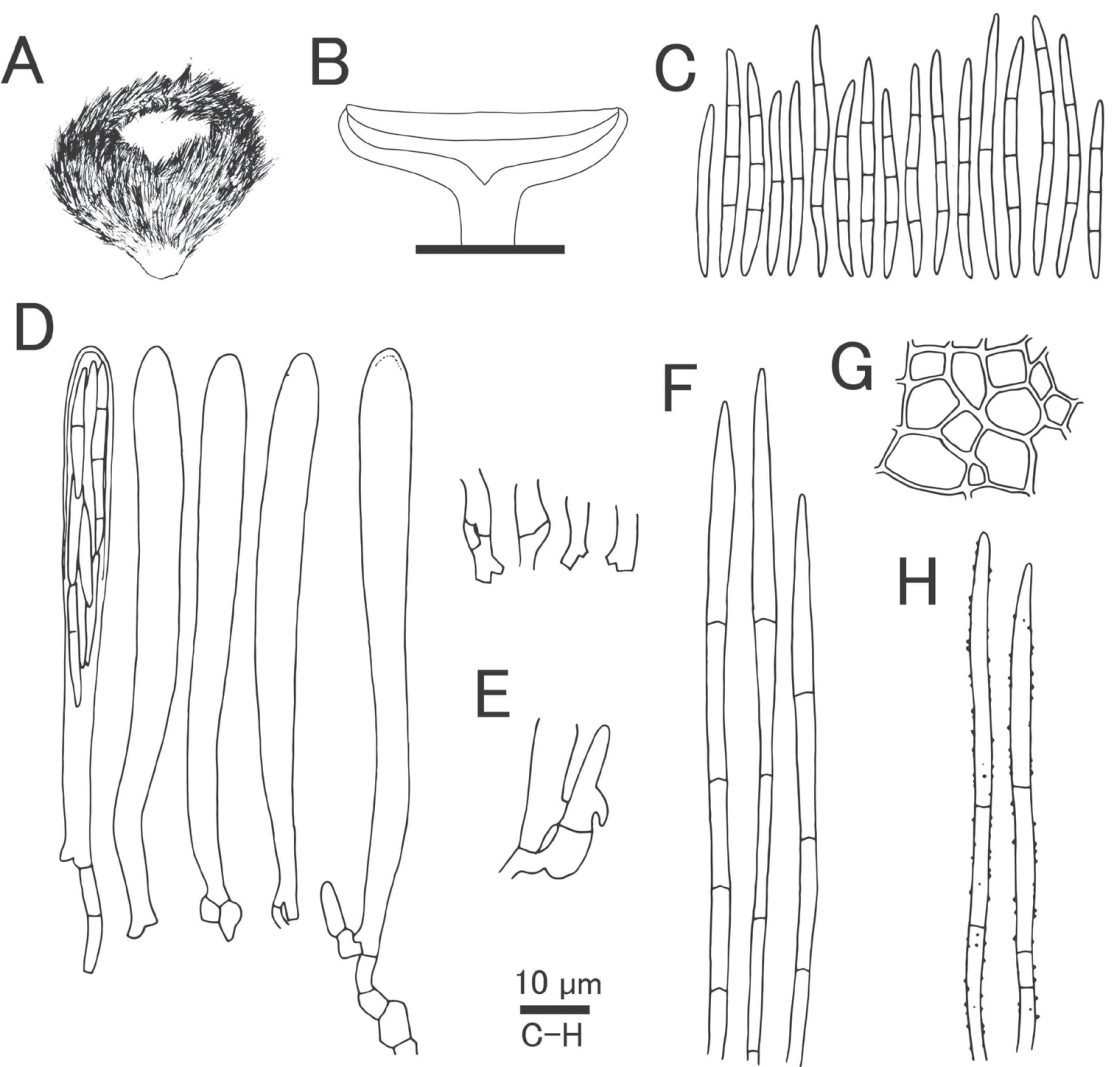
*Erioscyphellasasibrevispora*TNS-F-81401 (Holotype **A–D, F, G**). Lachnumnovoguineensevar.yunnanicumTNS-F-16642 (**E**) **A** apothecium **B** vertical section of an apothecium **C** ascospores **D** asci (with basal structures sometimes with perforation) **E** ascal base arising from a crozier with perforation **F** paraphyses **G** ectal excipular cells **H** hairs.

*Erioscyphellasasibrevispora* is closely related to L.novoguineensisvar.yunnanicum (TNS-F-16442, 16642) (Fig. 1) and occurs in the same habitats (that is, bamboo sheaths) but has shorter asci and ascospores. The ascal bases of the two species are very characteristic, in that they have croziers with perforations (Fig. 15G and Fig. 16E). In Lachnaceae, this type of crozier has only been reported in *Lachnellula* (Baral 1984). Additionally, both species exceptionally lack any hair materials in *Erioscyphella*.

The tropical species *E.bambusina* and Lachnumalbidumvar.americanum (Dennis) W.Y. Zhuang also occur on bamboo sheaths. However, compared with the present fungus, the former has smaller ascospores and filiform paraphyses (Dennis 1954), and the latter has extremely large asci and ascospores (Dennis 1960). In cool-temperate to subarctic zones, *L.asiaticum* and *Lachnumsasae* Raitv. occur on bamboo sheaths (Otani 1967; Raitviir 1985), but their ascospores are much shorter than those of the present fungus.

The wooly appearance and yellow disc of this species (Fig. 15A) resemble those of *Capitotricharubi* (Bres.) Baral; however, microscopic observations easily distinguish the two species.

### ﻿ Erioscyphella sinensis

Taxon classificationFungiHelotialesLachnaceae

(Z.H. Yu and W.Y. Zhuang) Sasagawa, Tochihara & Hosoya, comb. et stat. nov.

MycoBank No: 835709

≡ Lachnummapirianumvar.sinense Z.H. Yu and W.Y. Zhuang, Nova Hedwigia 74(3-4): 422 (2002).

#### Diagnosis.

Occurring on fallen leaves of of *Quercus* spp. or *Castanopsis* spp. in early summer and having needle-like ascospores.

#### Japanese name.

Shii-Kashi-hina-no-chawantake-modoki.

#### Specimen examined.

Japan, Ibaraki, Tsukuba, Mt. Tsukuba, 36.228539, 140.103504, ca 870 m, 23 Jun. 2007, on fallen leaves of *Castanopsissieboldii*, R.Sasagawa (TNS-F-16841). Japan, Ibaraki, Tsukuba, Amakubo, Tsukuba Botanical Garden, 36.101472, 140.110944, ca 20 m, 15 Jun. 2007, on fallen leaves of *C.sieboldii*, R.Sasagawa (TNS-F-16838). JAPAN, Tottori, Yonago, Yonago Castle, 35.42437, 133.325472, ca 50 m, 3 Jun. 2018, on fallen leaves of *C.sieboldii*, Y.Tochihara (TNS-F-81383).

#### Distribution.

China (Hainan, Yunnan; Yu and Zhuang 2003). Japan (warm-temperate zone).

#### Notes.

The present fungus was treated as *Lachnum* sp. 13 by Hosoya et al. (2010). This fungus occurs in the same habitats as *E.hainanensis*, but it is easily distinguished in having longer and needle-like ascospores. *Erioscyphellasinensis* resembles *L.mapirianum* in the shape of ascospores, but the two species are different in that *L.mapirianum* has long slender apothecial stipes, larger asci, longer ascospores, and wider paraphyses.

In the present study, we transferred this fungus to *Erioscyphella* and upgraded it from variety to species level, because this fungus is not phylogenetically related to ‘*L*’. *mapirianum* (Fig. 1). The presence of apical amorphous materials of hairs was confirmed in this study (Suppl. material 1: Fig. S2).

## Supplementary Material

XML Treatment for Erioscyphella boninensis

XML Treatment for Erioscyphella hainanensis

XML Treatment for Erioscyphella insulae

XML Treatment for Erioscyphella otanii

XML Treatment for Erioscyphella papillaris

XML Treatment for Erioscyphella paralushanensis

XML Treatment for Erioscyphella sasibrevispora

XML Treatment for Erioscyphella sinensis

## Supplementary materials

Supplementary material 1 Figure S1. ML trees Data type Image. Explanation note ML trees based on ITS (**A**), LSU (B), mtSSU (**C**) and RPB2 (**D**) constructed using MEGA X. Bootstrap values > 50% are indicated on branches and branches with MLBS > 70% are shown bold. https://binary.pensoft.net/file/643424This dataset is made available under the Open Database License (http://opendatacommons.org/licenses/odbl/1.0/). The Open Database License (ODbL) is a license agreement intended to allow users to freely share, modify, and use this Dataset while maintaining this same freedom for others, provided that the original source and author(s) are credited.Yukito Tochihara, Tsuyoshi Hosoya

Supplementary material 2 Figure S2. Hair apices Data type Image. Explanation note Hair apices of members of Clade **A***Erioscyphellaabnormis*TNS-F-32163 **B***E.abnormis*TNS-F-61773 **C***E.brasiliensis*TNS-F-46419 **D***E.sclerotii*TNS-F-26492 **E** ‘Lachnum’ mapirianumTNS-F-17245 **F** ‘Lachnum’ palmaeTNS-F-17567 **F1** Hair with resinous matters **F2** Hair with apical amorphous material **G** ‘Lachnum’ palmaeTNS-F-24600 **G1** Hair with a resinous matter **G2** Hair with apical amorphous materials **H***E.hainanensis*TNS-F-80371 **I***E.sinensis*TNS-F-80354. Mounted in CB/LA. Scale bars: 10 mm. Arrowheads show hair apical materials. https://binary.pensoft.net/file/643425This dataset is made available under the Open Database License (http://opendatacommons.org/licenses/odbl/1.0/). The Open Database License (ODbL) is a license agreement intended to allow users to freely share, modify, and use this Dataset while maintaining this same freedom for others, provided that the original source and author(s) are credited.Yukito Tochihara, Tsuyoshi Hosoya

Supplementary material 3 Figure S3. Result of the ASAP species delimitation analysis Data type Image. Explanation note The graph shows the distribution of ASAP scores according to partitioning results, and the phylogenetic tree shows the way of partitioning. https://binary.pensoft.net/file/643426This dataset is made available under the Open Database License (http://opendatacommons.org/licenses/odbl/1.0/). The Open Database License (ODbL) is a license agreement intended to allow users to freely share, modify, and use this Dataset while maintaining this same freedom for others, provided that the original source and author(s) are credited.Yukito Tochihara, Tsuyoshi Hosoya

Supplementary material 4 Figure S4. Result of the GMYC species delimitation analysis Data type Image. Explanation note Number with each node shows the support value that each cluster is an independent species. https://binary.pensoft.net/file/643427This dataset is made available under the Open Database License (http://opendatacommons.org/licenses/odbl/1.0/). The Open Database License (ODbL) is a license agreement intended to allow users to freely share, modify, and use this Dataset while maintaining this same freedom for others, provided that the original source and author(s) are credited.Yukito Tochihara, Tsuyoshi Hosoya

Supplementary material 5 Figure S5. ML best-scored phylogenetic tree based on concatenated dataset of ITS1, 5.8S, and ITS2 constructed by RAxML-NG Data type Image. Explanation note GenBank/UNITE accession number and TNS specimen number (if any) is shown for each taxon. MLBP > 50% were attached on branches. https://binary.pensoft.net/file/643428This dataset is made available under the Open Database License (http://opendatacommons.org/licenses/odbl/1.0/). The Open Database License (ODbL) is a license agreement intended to allow users to freely share, modify, and use this Dataset while maintaining this same freedom for others, provided that the original source and author(s) are credited.Yukito Tochihara, Tsuyoshi Hosoya

Supplementary material 6 Figure S6. Results of PTP species delimitation analyses Data type Image. Explanation note Number with each node shows the probability of the likelihood that each cluster is an independent species. Clusters showed by red branches are regarded as species. https://binary.pensoft.net/file/643429This dataset is made available under the Open Database License (http://opendatacommons.org/licenses/odbl/1.0/). The Open Database License (ODbL) is a license agreement intended to allow users to freely share, modify, and use this Dataset while maintaining this same freedom for others, provided that the original source and author(s) are credited.Yukito Tochihara, Tsuyoshi Hosoya
