# Supplementary figures and images for: ﻿Examination of the generic concept and species boundaries of the genus Erioscyphella (Lachnaceae, Helotiales, Ascomycota) with the proposal of new species and new combinations based on the Japanese materials
Source: MycoKeys. 2022 Feb 8;87:1–52. doi: 10.3897/mycokeys.87.73082 (PMC8847282; doi:10.3897/mycokeys.87.73082)

A

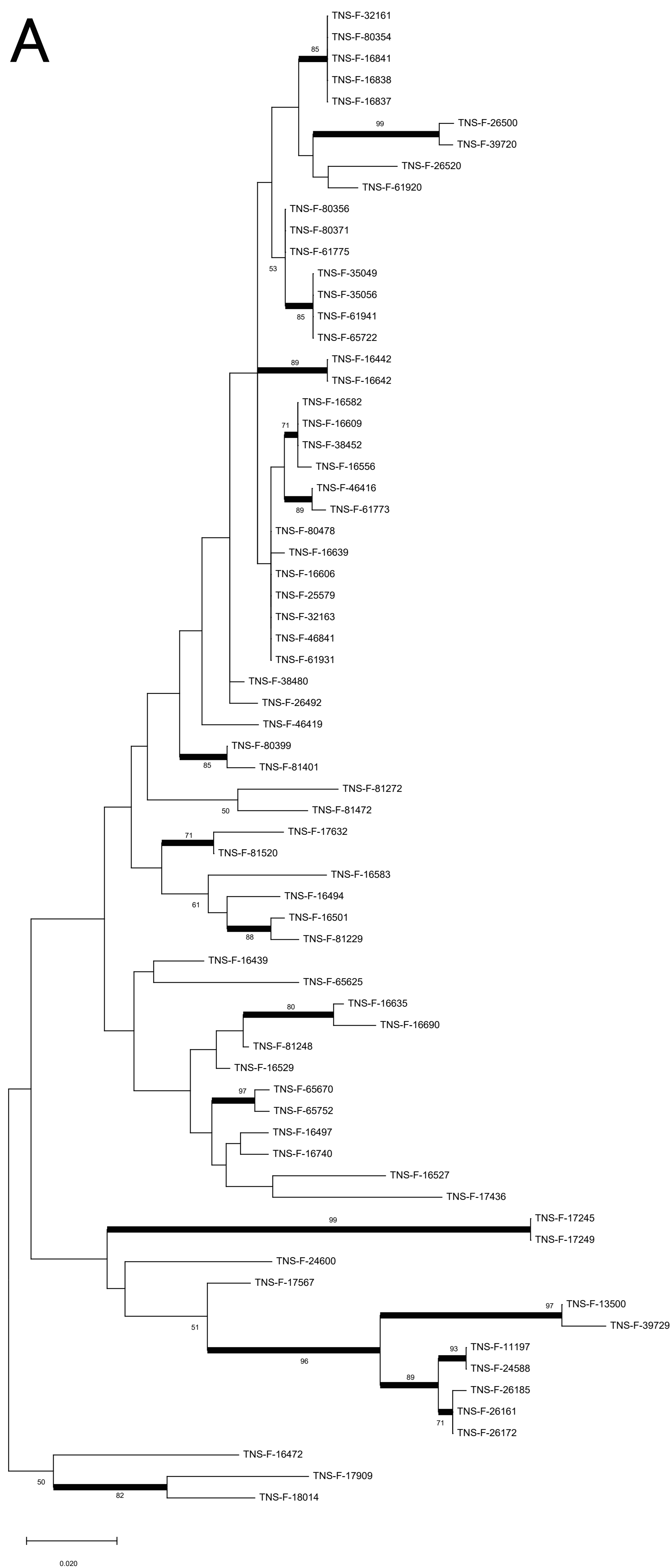

B

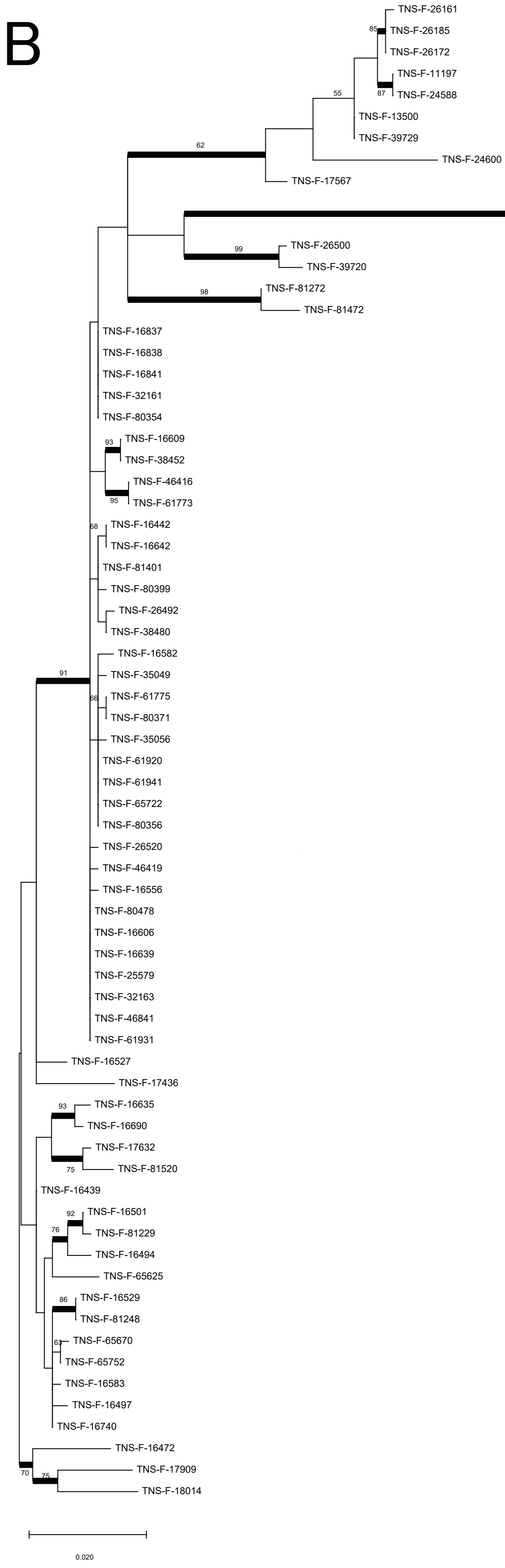

C

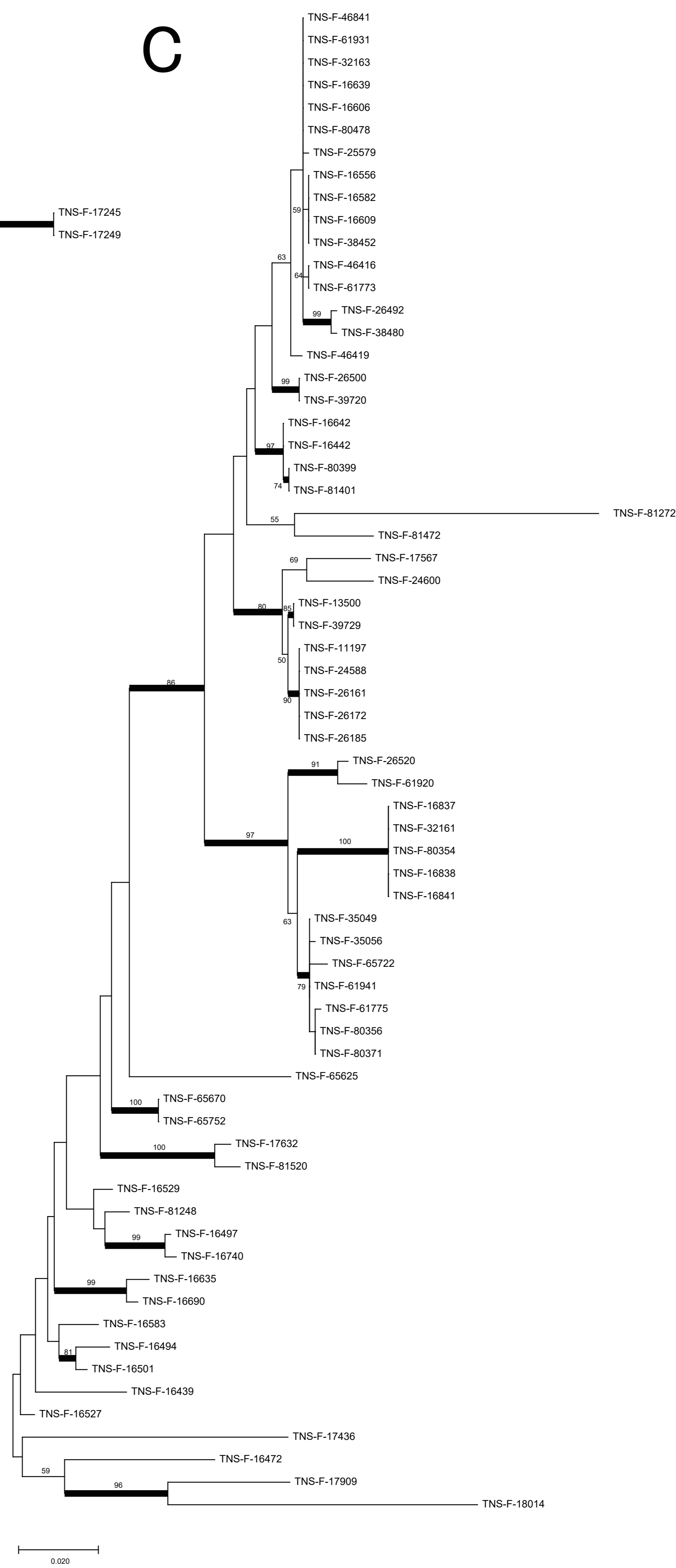

D

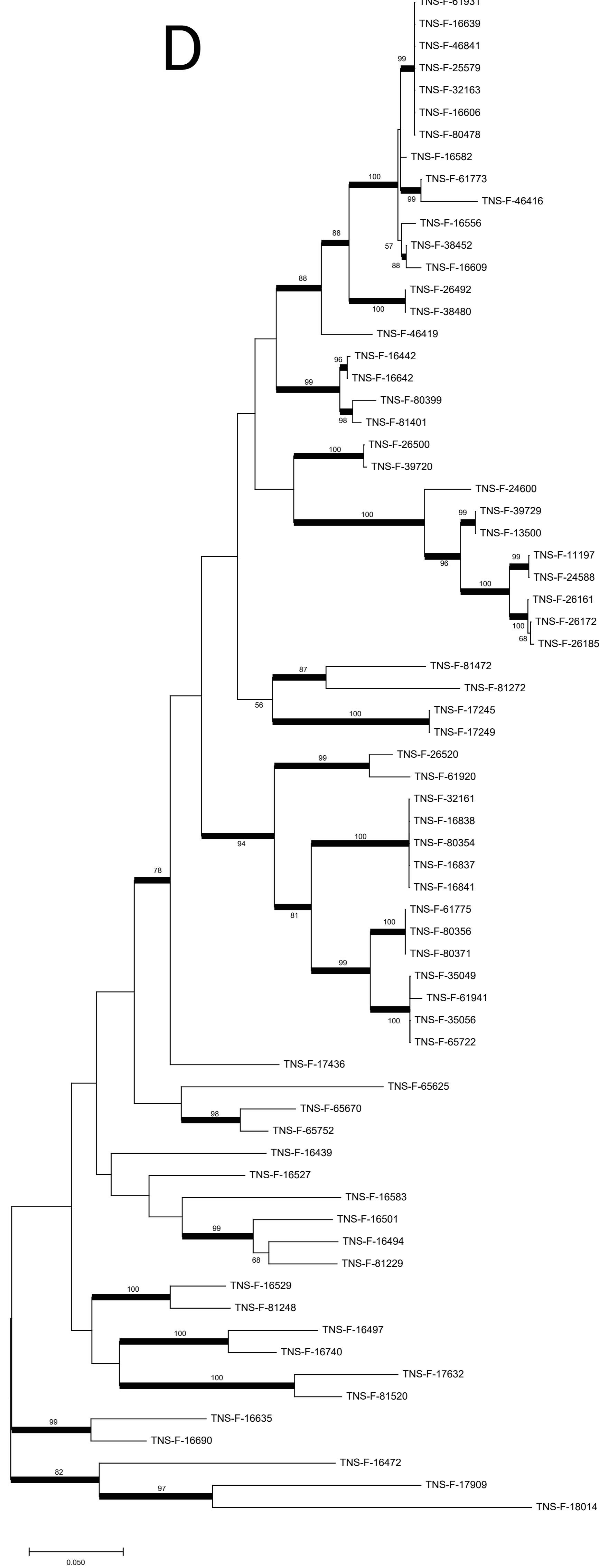

Supplement: Supplementary material 1 — Figure S1. ML trees [file mycokeys-87-001-s001.pdf]

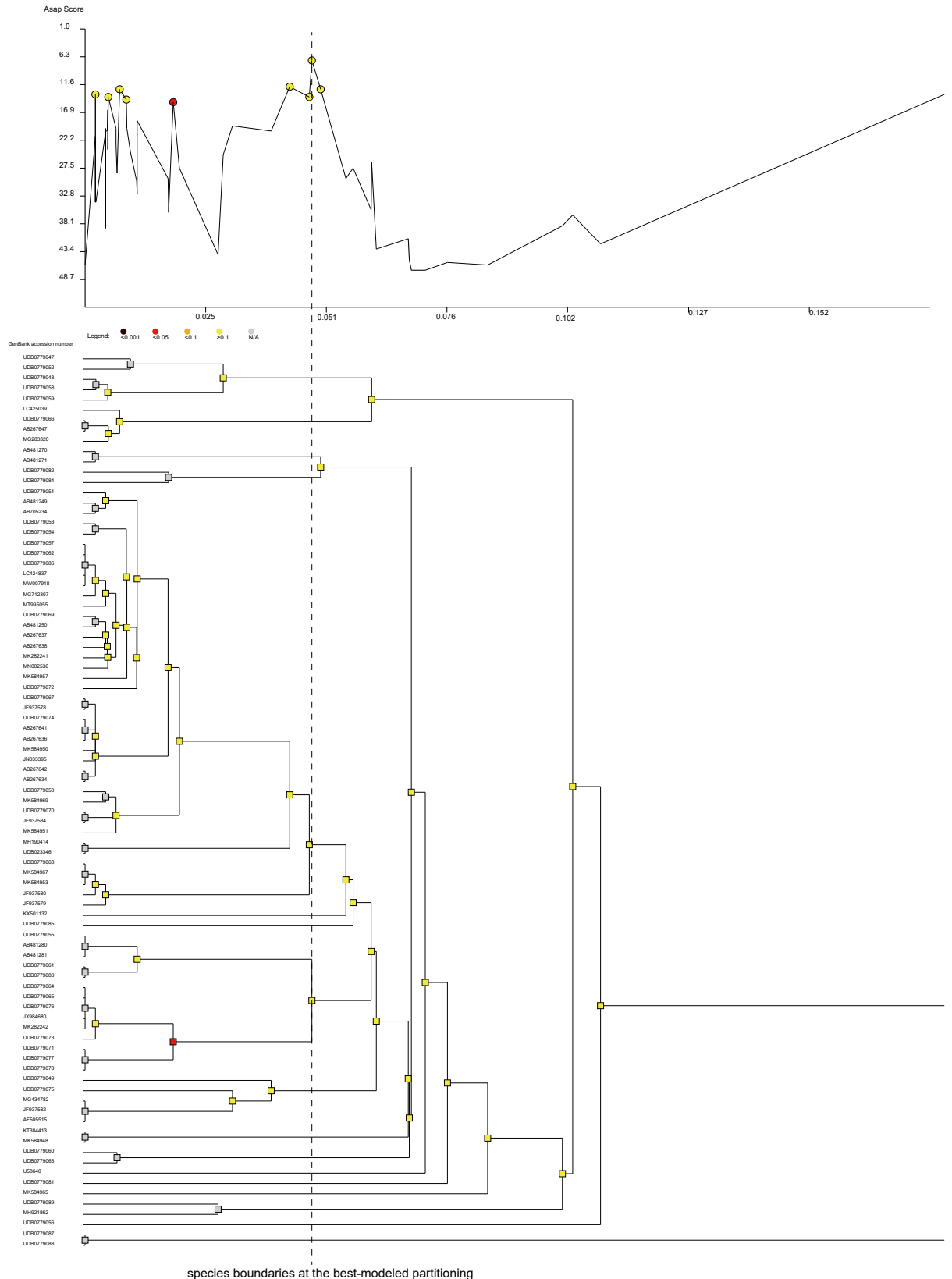

Supplement: Supplementary material 2 — Figure S2. Hair apices [file mycokeys-87-001-s002.pdf]

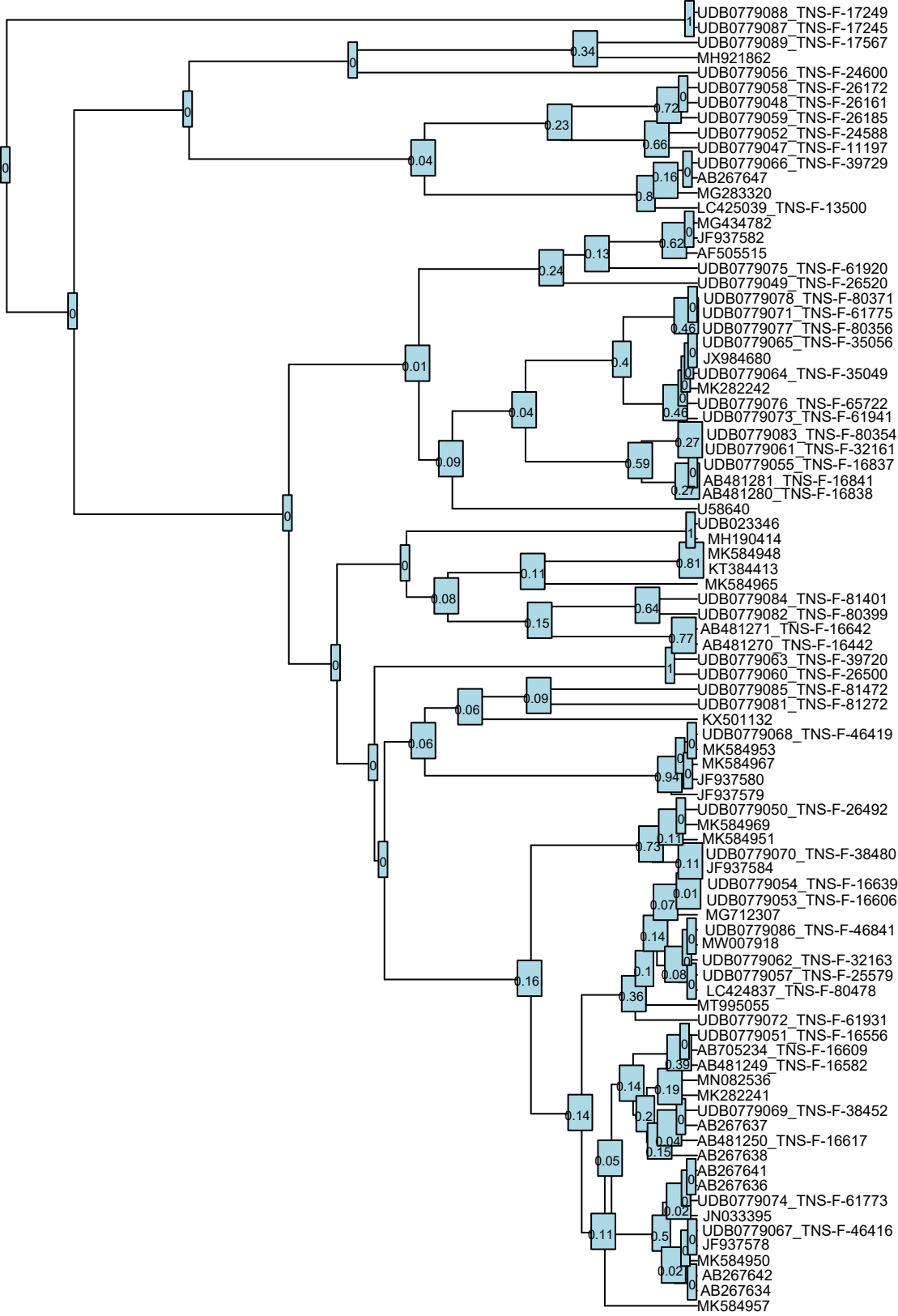

Supplement: Supplementary material 3 — Figure S3. Result of the ASAP species delimitation analysis [file mycokeys-87-001-s003.pdf]

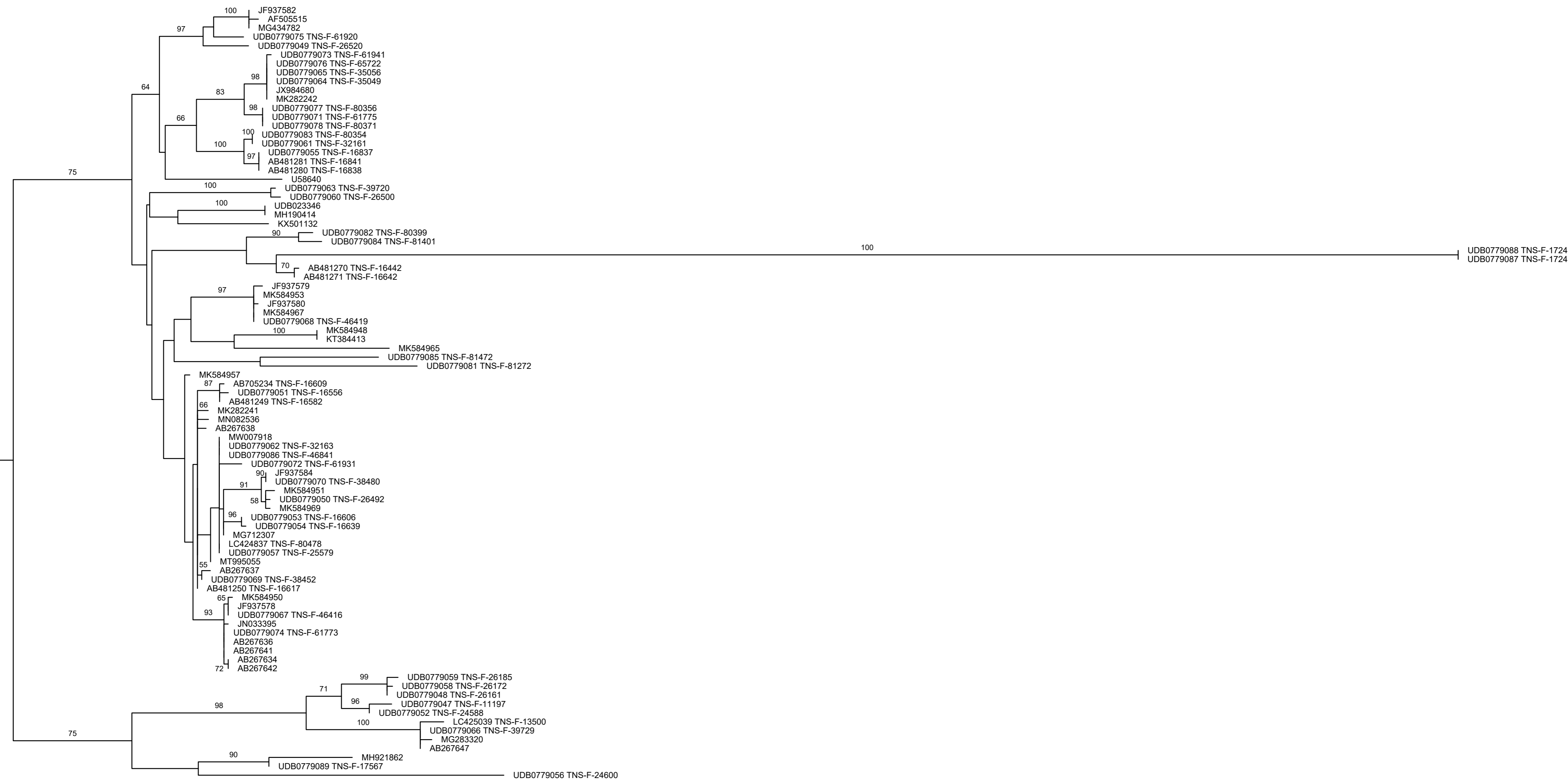

0.08

Supplement: Supplementary material 4 — Figure S4. Result of the GMYC species delimitation analysis [file mycokeys-87-001-s004.pdf]

Bayesian support

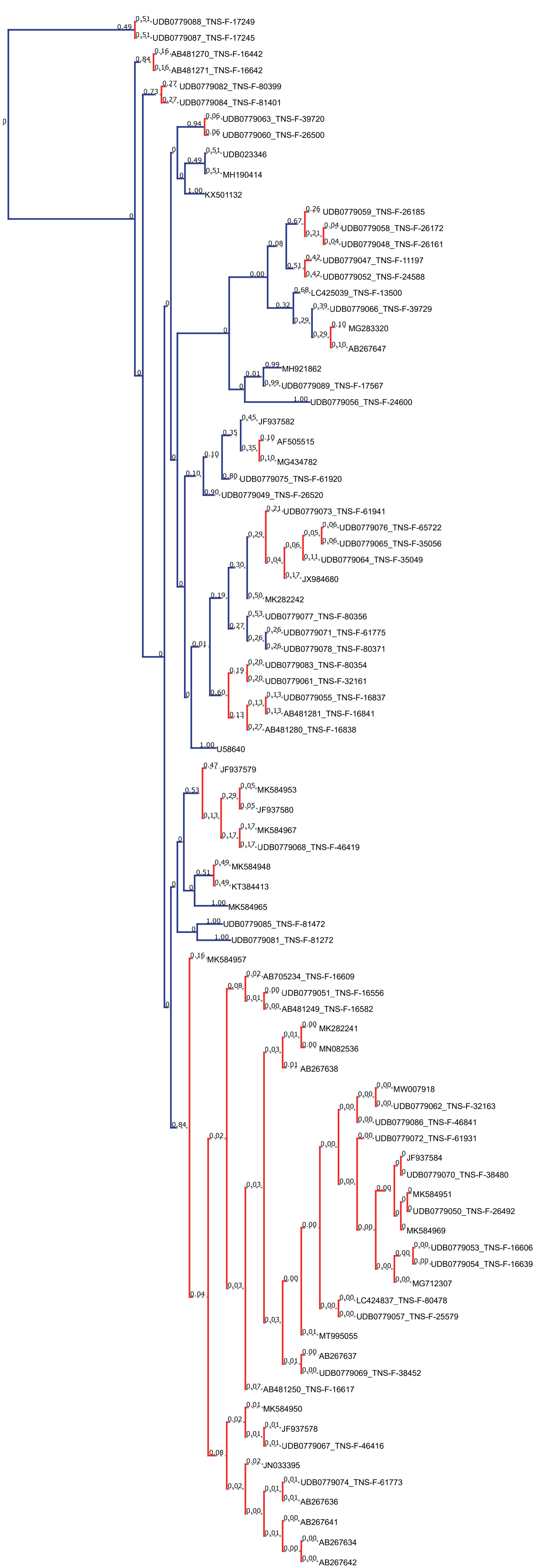

ML support

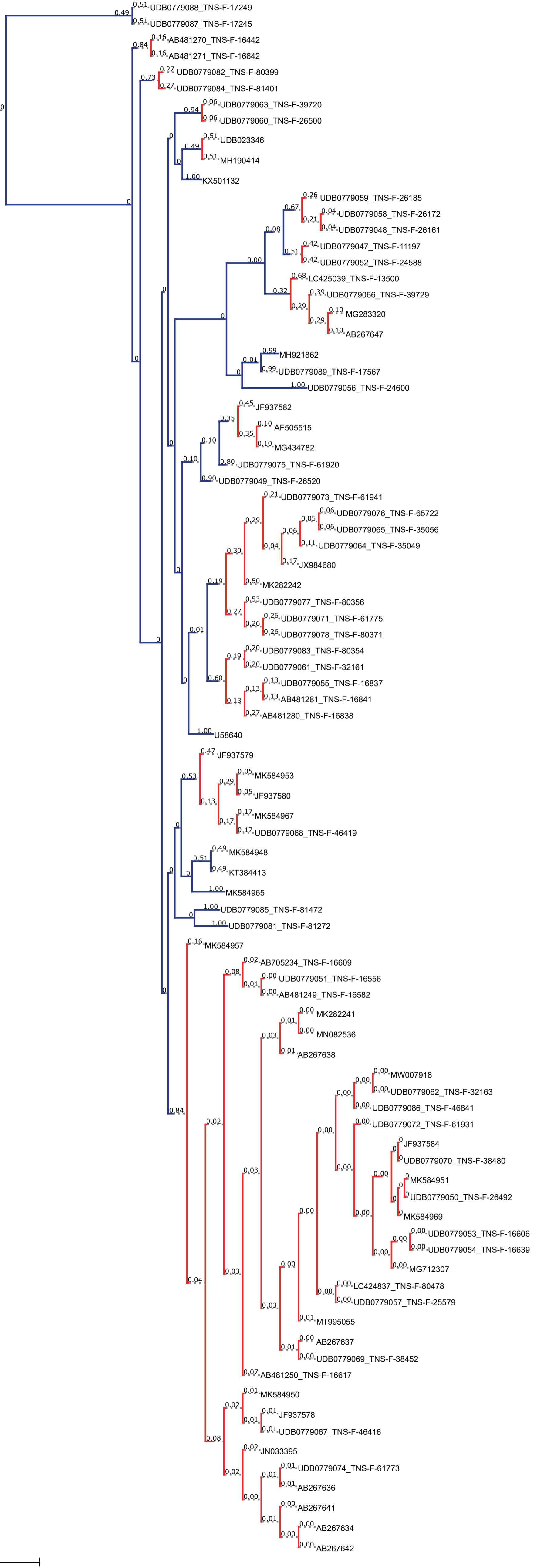

Supplement: Supplementary material 5 — Figure S5. ML best-scored phylogenetic tree based on concatenated dataset of ITS1, 5.8S, and ITS2 constructed by RAxML-NG [file mycokeys-87-001-s005.pdf]
